# Effect of HDL disk and LDL dimer presence on lipoprotein particle number determination and subclassification

**DOI:** 10.1007/s00216-026-06390-9

**Published:** 2026-02-27

**Authors:** Zsuzsanna Kuklenyik, Anna A. Ivanova, Lauren E. Drinkard, David M. Schieltz, Jeffrey I. Jones, Kevin Bierbaum, Christopher A. Toth, Michael S. Gardner, Bryan A. Parks, Michael Andrews, Jennifer D. Kusovschi, Jack A. Sultan, Antony Lehtikoski, Jon C. Rees, Wanda I. Santana, Victoria Connor, Eric C. Leszczynski, Robert W. McGarrah, Mark A. Sarzynski, William E. Kraus, John R. Barr

**Affiliations:** 1 Clinical Chemistry Branch, Division of Laboratory Sciences, Centers for Disease Control and Prevention, 4770 Buford Highway, Atlanta, GA 30341 USA; 2 Duke Molecular Physiology Institute, Duke University School of Medicine, Duke University, Durham, NC 27701 USA; 3 University of South Carolina, 921 Assembly Street, Columbia, SC 29201 USA

**Keywords:** Apolipoprotein composition, Lipoprotein particles, Lipoprotein particle numbers, HDL, LDL, Lipoprotein particle size measurement, Asymmetric-flow field-flow fractionation, Lipid homeostasis

## Abstract

High-density and low-density lipoproteins (HDL and LDL) are established analytical targets for diagnosis and risk stratification of numerous chronic diseases. This study investigates potential sources of bias in lipoprotein particle counting (HDL-P and LDL-P), focusing on the most atheroprotective small-HDL and most pro-atherogenic small-LDL. Plasma samples were fractionated using asymmetric-flow field-flow fractionation (AF4), coupled with hydrodynamic size measurement and comprehensive liquid chromatography-tandem mass spectrometry (LC-MS/MS) analysis of each fraction. Concentration-size profiles were deconvoluted into 10 HDL and 8 LDL Gaussian subspecies. Molecular volume ratios were used to evaluate proposed particle models, providing evidence for the presence of s-HDL disk and s-LDL dimers, as sources of bias in calculated HDL-P and LDL-P when spherical particle geometry is assumed. Matching apoA1/HDL-P and apoB/LDL-P to consensus values enabled correction of mass diameters (k*d_m_), with k≈1.2 for s-HDL disks, and k≈0.8 for s-LDL dimers. These corrections resulted in closer agreements of the calculated HDL-P and LDL-P values with those reported by nuclear magnetic resonance spectroscopy (n = 666). After corrections, average AF4-LC-MS/MS versus NMR differences decreased from 20 to 4.9% for HDL-P and from −47% to −5.7% for LDL-P; x-y correlations improved from 0.83 to 0.85 and 0.51 to 1.03, respectively; and both platforms showed a dominant presence of s-HDL and s-LDL subclasses. Overall, the results support standardization of clinical methodologies for lipoprotein subclass measurement around apoA1 and apoB, and harmonization of HDL and LDL subclass definitions based on composition and structural characteristics rather than strictly enforced size cutoffs.

**Graphical abstract:**

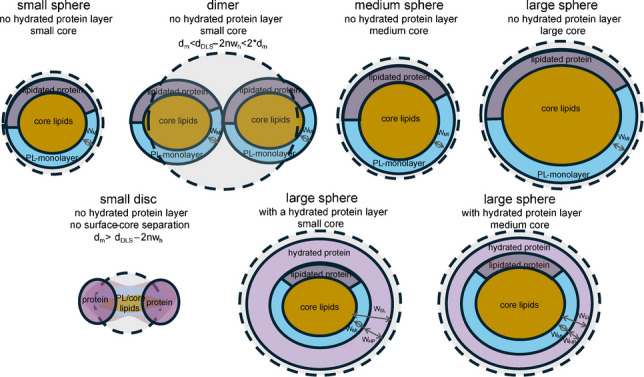

**Supplementary Information:**

The online version contains supplementary material available at 10.1007/s00216-026-06390-9.

## Introduction

Lipoproteins are nanometer-scale particles composed of lipids and proteins and are conceptualized as spherical, quasi-spherical, or discoidal structures in which proteins and phospholipids (PL) are distributed on the surface, while the relatively non-polar cholesteryl esters (CE) and triglycerides (TG) reside in the core [1, 2]. Free cholesterol (FC) partitions between the surface and core lipid phases [3]. Amphipathic PL molecules form a surface monolayer that serves as an anchor for numerous proteins and other biomolecules. Among these proteins are apolipoproteins (apos), which contain amphipathic α-helix and β-sheet domains that embed within the PL monolayer, whereas non-amphipathic domains dynamically protrude from the surface or interact only with the polar PL headgroups [4].

Traditionally, lipoproteins are classified by density into high-, low-, and very-low-density classes (HDL, LDL, and VLDL) [5, 6]. In this hierarchy, decreasing density among the major lipoprotein classes corresponds to increasing particle size and a decreasing proportion of protein mass relative to lipid content. The distinction between HDL and non-HDL lipoproteins (LDL and VLDL) is further reflected by the predominant presence of apolipoprotein A1 (apoA1) in HDL and apolipoprotein B-100 (apoB) in LDL and VLDL [5, 6].

On scales of density, size, and electrostatic charge, numerous subspecies exist within the major lipoprotein classes. Using different physical properties, loosely equivalent (~) subclass nomenclatures have been established. The two main HDL subclasses are commonly distinguished as HDL3 ~ dense ~ small ~ α3/α4 versus HDL2 ~ buoyant ~ large ~ α2; and the two main LDL subclasses are described as dense ~ small ~ more-negatively charged versus buoyant ~ large ~ less-negatively charged [7–9]. Despite efforts to harmonize the physical cut-points between these main subclasses across methods, limited comparability has been achieved in terms of relative concentration patterns and disease risk stratification [5, 7, 10, 11]. The lack of suitable reference materials remains the primary obstacle toward better harmonization. Nonetheless, associations between the physical properties of lipoprotein subclasses and atherogenicity remain a compelling area of investigation, mostly for small (s-), medium (m-), and large (l-) subclasses [11–15].

According to current theories, s-HDL is atheroprotective by being a potent mediator of cholesterol efflux from peripheral cells and macrophages [11]. The gained FC content of s-HDL is then converted to CE, resulting in an increase in particle size to m-HDL and l-HDL. In contrast, nascent TG-rich, apoB-containing particles decrease in size by lipolysis and become LDL before receptor-mediated reuptake. Delayed clearance causes prolonged circulation, resulting in more s-LDL compared to m/l-LDL production, and an increased probability of oxidation of the lipid cargo. Oxidation may also promote aggregation and fusion of s-LDL at the arterial wall, triggering a cascade of inflammatory responses and atherosclerotic plaque formation [16–18].

As nanoparticle-like entities, lipoproteins are ideally quantified by lipoprotein particle number (Lp-P), expressed in moles per original sample volume (before fractionation) [19]. Measurement of Lp-P has the potential to better characterize many lipid metabolism-related disease states compared to traditional lipid profiling based on total cholesterol content (TC = FC + CE) of HDL and LDL [20]. Gas-phase ion-mobility (IM) platforms generate Lp-P size profiles, calibrated with gold nanoparticles, but fragile lipoproteins may lose loosely bound proteins during electrospray ionization, thereby distorting results [21–23]. In contrast, proton (^1^H) nuclear magnetic resonance (^1^H-NMR) spectroscopy offers a simpler, gentler approach to Lp-P measurement, requiring only sample dilution. This NMR method estimates lipid concentrations (FC, CE, TG, PL) and particle size based on methyl or methylene signal intensities and magnetic susceptibility for acyl and alkyl chains of lipids [24, 25].

Segrest et al. [26] and Schaefer et al. [27] introduced a “volumetric approach” to calculate Lp-P. This approach requires preparative fractionation followed by lipoprotein constituent concentration and size measurements in the fractions. Total partial molecular volumes of the lipoprotein components in the fractions are then summed together. This total is then divided by the estimated partial specific volume of the particles. The volumetric approach is labor intensive and not feasible for clinical application, but its main advantage is its metrological traceability to purified and value-assigned PL, FC, CE, TG, and protein materials. Unlike IM or NMR platforms, it does not rely on assumptions about equivalency between lipoproteins and gold nanoparticle standards or proprietary software algorithms. Furthermore, measuring all major lipoprotein constituents allows subclass definitions based on relative compositional differences, instead of procedure- or technique-biased physical properties. Therefore, the volumetric approach is an important tool for better understanding lipoprotein particle count-size-composition-function relationships.

The primary objective of this study was to investigate potential biases in determining Lp-P for HDL and LDL subclasses using the volumetric approach. In a prior study, we applied this method and assessed its accuracy using per-particle apoA1 and apoB estimates, but we observed deviations from established consensus values (2–3 apoA1/HDL and 1 apoB/LDL) without a clear explanation. Here, as before, we fractionated samples using asymmetric-flow field-flow fractionation (AF4), and in each fraction we measured average particle size by dynamic light scattering (DLS), and quantified fraction concentrations via liquid chromatography-tandem mass spectrometry analysis (LC-MS/MS) [28, 29]. To enhance size resolution and Lp-P accuracy, we deconvoluted lipid and protein concentration-size profiles into theoretical Gaussian subspecies and introduced a shape correction factor (*k*) to account for structural variations, such as discoidal HDL and LDL dimers. Our results allow addressing key challenges of Lp-P measurement, including biases in Lp-P and composition estimates from continuous versus deconvoluted size profile data, and discrepancies between lipoprotein mass size and hydrodynamic size due to deviations from spherical geometry.

## Methods

### Sample information

In this study, we analyzed 666 human plasma samples from the CATHGEN archive, collected between 2004 and 2007 [30]. These samples were collected from participants during coronary artery catheterization procedures. All participants fasted ≥ 8 h per CATHGEN biorepository protocol. Blood was drawn into EDTA-containing tubes, immediately placed on ice, and centrifuged within 30 min at 4 °C to separate plasma. Aliquots were stored at −80 °C with no more than one thaw–refreeze cycle until analysis. Participant characteristics, including age (63 ± 12 years), BMI (30 ± 7 kg/m^2^), sex distribution (61% male), and prevalence of coronary artery disease (66%), diabetes (31%), hypertension (67%), and smoking (49%) are summarized in Supplementary Table S1. All samples were de-identified, and the use of the CATHGEN data archive was approved by the Duke Health Institutional Review Board.

### NMR analysis

Plasma lipoprotein subclass measurements were obtained using ^1^H-NMR spectroscopy on a Vantera® clinical analyzer (LabCorp, Morrisville, NC). Lipoprotein particle concentrations and subclass distributions were derived using the LipoProfile-4 deconvolution algorithm, which models the composite methyl signal region of the NMR spectrum to quantify total and subclass-specific HDL-P and LDL-P. The proportionality of the deconvoluted methyl and methylene signal intensity patterns of HDL, LDL, and VLDL subclasses was validated using standard materials obtained by ultracentrifugation, size exclusion chromatography (SEC) fractionation, and size characterization by gradient gel electrophoresis (GGE) [31, 32]. Accuracy of total high-density and low-density lipoprotein particle numbers (HDL-P and LDL-P) on the NMR platform was further validated by comparison with apoA1 and apoB concentrations measured by independent methods [33, 34].

### Asymmetric-flow field-flow fractionation (AF4)

AF4 is a widely adopted technique in nanoparticle science, also applied for separation of lipid particles [35–37]. We applied AF4 with minor modifications as described in previous publications [28, 29]. The theoretical principles of size separation by AF4 are based on the fundamentals of Brownian diffusion and laminar flow dynamics. Retention time in the channel is governed by the channel thickness (w_*ch*_) and particle diffusion coefficients (D_*i*_), (w_*ch*_)^2^/D_*i*_. Retention time also depends on the channel length (L) and the ratio of perpendicular cross-flow (F_x_) to parallel channel-flow (F_ch_), approximated as *ln(1* + *F*_*x*_/*F*_*ch*_*)*. At the end of the channel, the channel-flow is split into a slot-flow (laminal layers of buffer, F_slot_) and a detector-flow (particle-carrying laminar layers, F_det_). In this study, we injected 50 µL plasma into the AF4 channel during an 8-min injection/focusing period. Method parameters included: w_*ch*_ = 0.45 mm, F_x_ = 3.2–0.2 mL/min decrease in 100 min, F_det_ = 0.1 mL/min, F_slot_ = 0.35 mL/min, and F_ch_ = 0.45 mL/min (F_ch_ = F_det_ + F_slot_) (Figure S1A). We collected 38 fractions (250 μL each) between 5 and 95 min during the elution of HDL and LDL particles, followed by 2 fractions (500 μL each) of VLDL during the first 10 min of channel purge (F_x_ = 0 mL/min). The carrier buffer consisted of 10 mM sodium bicarbonate and 75 mM NaCl (pH 7.4). Inter-day method variation is shown by average (SD) profiles for the quality control pool in Figure S1C. One freeze-thaw cycle had minimal effect on profiles’ reproducibility, compared to inter-day method variations and differences between individuals even if analyzed in the same batch (Figure S1E).

### Hydrodynamic size measurement

The average hydrodynamic diameter (*d*_*DLS*_) in each fraction was measured using DLS with a Dynapro plate reader (Wyatt Technologies, Santa Barbara, USA). When AF4 is applied with constant cross-flow, a fundamentally linear relationship exists between hydrodynamic size and retention time (elution time). To minimize elution volume and the number of fractions, we applied a cross-flow gradient with an exponential decay, resulting in an exponentially increasing size-versus-fraction-number relationship (Figure S1 A–B). For each sample run, a y = a + bx + c*(x−d)^2^ function was optimized with weights applied at fraction numbers corresponding to the size-profile maxima of albumin (7.4 nm) and transferrin (8.5 nm) (Table S2). Repeated fractionation of a quality control (QC) pool, fractionated alongside sets of five unknown samples during the study, demonstrated ± 5–7% size measurement reproducibility (Figure S1B).

### LC-MS/MS analysis of proteins and lipids

The optimization and validation of the online trypsin digestion-coupled LC-MS/MS proteomics and targeted lipidomics methods have been previously published elsewhere [38–41]. The protein and lipid class concentrations in the fractions were determined using a dilution series of a plasma pool. The plasma pool was previously value-assigned using certified pure protein standards or serum reference materials [38]. The percentage variation in measured protein and lipid concentrations in whole plasma ranged from 5 to 20%. Concentration precision in the fractions varied across the size distribution profile of specific analytes, from 15 to 30%, with higher variation near the peak baseline (Figure S1C).

### AF4 recovery correction

The AF4-Recovery for each analyte was calculated by summing the mole amounts in all fractions, [Concentration of analyte in fraction] × [Fraction volume], including fractions collected during purge containing VLDL particles, and divided by the amount measured in the unfractionated plasma injected into the AF4 system, Eq. 1.

1 $$AF4\_\mathrm{Recovery}=\frac{\sum_{n=1}^{40}\left[\mathrm{Concentration}\;\mathrm{of}\;\mathrm{analyte}\;\mathrm{in}\;\mathrm{fraction}\;\right]\ast\lbrack\mathrm{Fraction}\operatorname{Volume}\rbrack}{\left[\mathrm{Total}\;\mathrm{concentration}\;\mathrm{in}\;\mathrm{plasma}\right]\ast\lbrack\mathrm{volume}\;\mathrm{of}\;\mathrm{plasma}\;\mathrm{injected}\;\mathrm{into}\;\mathrm{the}\;\operatorname{AF}4\;\mathrm{channel}\rbrack}$$

The fraction concentrations were corrected with the AF4 channel recovery, Eq. 2

2 $$\left[\mathrm{equivalent\;fraction\;concentration}\;\right]=\frac{\left[\mathrm{Concentration\;in\;fraction}\right]\ast\left[\mathrm {Fraction\;Volume}\right]}{\left[\mathrm{Volume\;of\;plasma\;injected\;in\;to\;the\;AF4\;channel}\;\right]}\ast\frac1{\mathrm{AF4\_}\mathrm{Recovery}}[\mathrm{mole/L}]$$

Since TG recovery was the lowest (30–50%), most likely due to lower recovery of the most TG-rich large VLDL particles, TG recovery was based on the apoA1 recovery (70–90%) for HDL fractions and apoB recovery (50–70%) for LDL fractions. Although the VLDL fractions, collected at zero cross-flow during the purge, were analyzed, the VLDL fraction concentrations have a role only in the determination of the AF4 recovery for this study.

### Deconvolution

Since the hydrodynamic size measurements were unique to each fraction and each sample run, for visual evaluation of average concentration profiles we performed size binning. The size scale of the AF4 profiles was binned into 0.75, 1.5, and 3 nm increments in the HDL, LDL, and VLDL regions, respectively (corresponding to 1–2 fractions per bin), followed by summing the fraction concentrations by size bin (Figures S3–S8). However, this binning approach reduced size resolution.

As an alternative to binning that preserves size resolution, deconvolution was applied. We deconvoluted the size profiles for each analyte and sample, such that the sum of the Gaussian subspecies values had to match the measured concentration for each sized size-resolved fraction (without size scale binning). In the HDL and LDL size ranges, the AF4 profiles for each protein and lipid class were deconvoluted into *n* = 15 Gaussian components using Eq. 3.

3 $$f\left(Q_i,\Delta,w_{1/2},s\right)=\sum\nolimits_{i=1}^{n=15}Q_i\ast e^\frac{{-(d_{DLS}-s+i\ast\Delta)}^2}{2\ast w_{1/2}^2}$$

where the *Q*_*i*_ constants determine the intensity of each Gaussian peak, *d*_*DLS*_ is the measured hydrodynamic size of the fractions (nm), *i* is the number of increments from the starting size *s* (nm), Δ is the size increment between adjacent Gaussian peaks, and *w*_½_ is the half-peak width. The Gaussian parameters were set as follows: *s* = 6 nm, *w*_1/2_ = 0.6 nm, and Δ = 0.75 nm for HDL; and *s* = 13 nm, *w*_1/2_ = 1.5 nm and Δ = 1.5 nm for LDL. The sizes of Gaussian components with above experimental baseline were as follows: for HDL 7.5, 8.25, 9, 9.75, 10.5, 11.25, 12, 12.75, 13.25, and 14 nm; and for LDL 19, 20.5, 22, 23.5, 25, 26.5, 28, and 29.5 nm. Further details of the deconvolution procedure are provided in supplementary information.

The concentrations of individual Gaussian subspecies were derived from the optimized *Q*_*i*_ values, calculating the area under each Gaussian peak using Eq. 4. Hereafter, we refer to these Gaussian peaks and the corresponding concentrations as HDL and LDL subspecies, expressed as plasma concentration (nmol/L).

4 $$\left[Deconvoluted\;Gaussian\;subspecies\;concentration\right]=Q_i\ast\left(\pi\right)^{1/2}[\mathrm {mole/L}]$$

### Correction for deviation from spherical particle shape

**Fig. 1:**
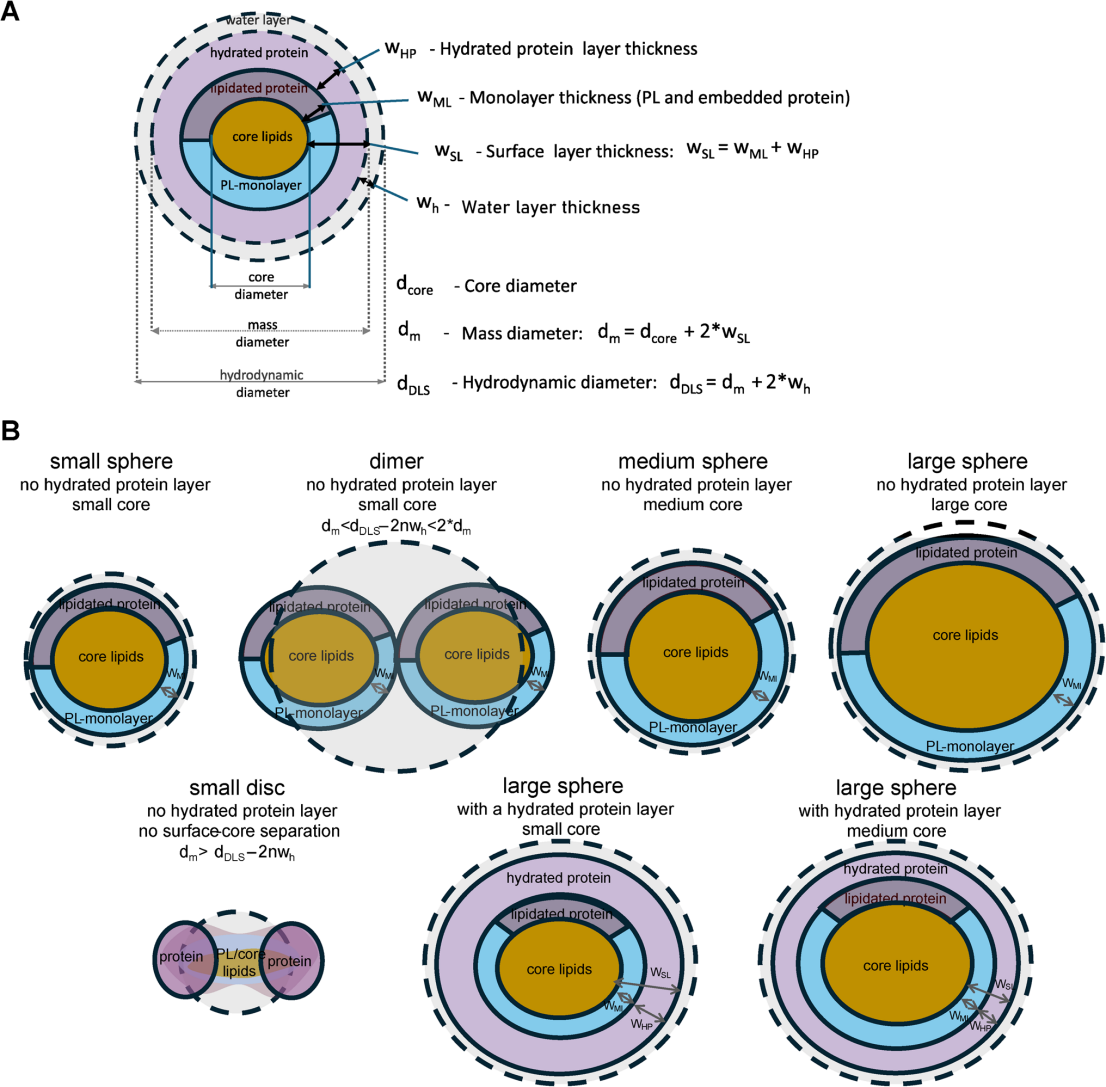
Schematic representation of lipoprotein models and the volumetric parameters. **A** Default model with hydrated protein layer. Water layer (gray), hydrated protein layer (light purple), PL-monolayer (light blue), lipidated protein layer (dark purple), and PL-monolayer (orange) are depicted. **B** Deviations from the default model (disks, dimers, and varying hydrated protein layer thickness). Note: The models allow prediction of deviation from d_m_ estimated based on spherical particles (d_DLS_ − 2 w_h_). HDL disks formation causes d_m_ > (d_DLS_ − 2 w_h_), and LDL dimer formation causes d_m_ < (d_DLS_ − 2 w_h_) < 2 dm

In this work, we conceptualize lipoproteins as illustrated in Fig. 1. The measured hydrodynamic diameter d_DLS_ represents the size of an equivalent imaginary sphere exhibiting the same hydrodynamic friction as the actual lipoprotein particle. This degree of friction depends on the number (n) of theoretical water layers (w_h_) dragged along with the particle due to Brownian motion, where the thickness of each (w_h_) is ~ 0.3 nm.

According to the default model in Fig. 1A, the measured d_DLS_ is the sum of the mass diameter (d_m_) and the thickness of the water layers. Therefore, d_m_, the diameter of a sphere with the same partial specific volume as the sum of the partial specific volumes of all proteins and lipids on the particle, can be estimated from d_DLS_.

5 $${d}_{m}= {d}_{DLS}- 2n*{w}_{h}$$

To correct for deviation from spherical shape, a *k* factor was incorporated which reflects the difference between the d_m_ of a non-spherical particle relative to a spherical particle.

6 $${d}_{m}= {k*(d}_{DLS}- 2n*{w}_{h})$$

With respect to particle shape and mass distribution, small discoidal HDL particles exhibit protein mass preferentially distributed toward the edge of the disk. Based on similarity to idealized geometric shapes, small HDL particles can be approximated as hollow disk or torus, while LDL dimers can be approximated as cylinders (Figure S9). Thus, d_m_ of both HDL disks and LDL dimers is underestimated when calculated from d_DLS_ − 2n*w_h_ by assuming a spherical shape with the same d_DLS_. Shape correction with k > 1 is necessary for both discoidal HDL (hollow disk) and dimeric LDL (cylinder). However, for the spherical LDL monomer inside the dimer, approximately one-half of the LDL-dimer correction is necessary, i.e., corresponding to k < 1.

The k-correction can be independently estimated, with apoA1/HDL-P and apoB/LDL-P calculated assuming a spherical shape from the experimental data and the consensus values of 2–4 apoA1 per HDL and 1 apoB per LDL, using Eqs. 7 and 8.

7 $$\left[k-correction\;factor\;for\;HDL\right]=\sqrt[3]{\frac{\lbrack apoA1\;perHDL\;by\;consensus\rbrack}{\lbrack calculated\;apoA1/HDL-P\;for\;spherical\;shape\rbrack}}$$

8 $$\left[k-correction\;factor\;for\;LDL\right]=0.5\ast\sqrt[3]{\frac{\lbrack apoB\;perLDL\;by\;consensus\rbrack}{\lbrack calculated\;apoB/HDL-P\;for\;spherical\;shape\rbrack}}$$

### Calculation of particle number and composition

To calculate particle volume estimates, we first converted the molar analyte concentrations to “molecular volume concentrations”, by multiplying molar concentration by partial specific volume. Partial specific volumes were adopted from the literature [2, 26, 27]. The sum of the lipoprotein-carried “molecular volumes concentrations” was divided by the particle nm^3^ volume based on the estimated d_m_, yielding molar concentration of lipoproteins (Lp-P, or HDL-P and LDL-P). As demonstrated by a simulation in Figure S9, the Lp-P calculations were affected most strongly by the d_m_. Relative to the variation and bias introduced by (d_m_)^3^ used to calculate Lp particle volume, the literature-derived partial specific volumes of lipid and protein molecules had negligible impact on the calculated Lp-P accuracy. To maximize the accuracy of the d_DLS_ measurements, it was essential to isolate and fractionate samples in small volume increments, i.e., by collection of 40 fractions from each plasma sample.

### Evaluation of statistically significant differences

JMP software (versions 15 and Pro 16, SAS Institute) was used for all calculations. The criterion of statistically significant differences was p-value < 0.05. Since mean values were calculated from 666 samples, the approximate level of statistical significance can be evaluated by looking at the separation between the 5th and 95th confidence interval error bars used in our figures. The significance of correlations was assessed with x-y plots based on slopes and R^2^ values.

## Results

### Models and assumptions

Our conceptual default model of spherical lipoproteins is shown in Fig. 1A, where the dashed outer circle represents the measured hydrodynamic size (d_DLS_) that differs from the mass diameter (d_m_) due to the water layer thickness (w_h_). Proteins in the monolayer are depicted as a continuous segment within the monolayer. In native particles, the amphipathic protein domains are expected to spread laterally while contributing to the monolayer thickness (w_ML_). Hydrated protein domains may extend irregularly outward from the monolayer, a feature reflected in the model by an average hydrated protein layer thickness positioned above the monolayer (w_HP_). The total surface layer thickness (w_SL_) comprises both the monolayer and the hydrated protein layer.

Possible deviations from the default model are shown in Fig. 1B, which illustrates our central underlying concept that at similar w_h_ and w_ML_, the core/surface volume ratio depends on d_DLS_ and w_SL_, whereas the core-lipid/surface-lipid volume ratio depends on the d_DLS_ and the contribution of the embedded protein domains to the monolayer volume (%protein). Additionally, the formation of discoidal HDL and LDL dimer particles is expected to disrupt the proportional relationship between the measured d_DLS_ and the estimated d_m_, necessitating a k*d_m_ correction in Eq. 6.

Consideration of the conceptual models based on experimental data relies on several important assumptions. (1) Size-based separation under physiological buffer conditions preserves particle composition during the AF4 process. (2) Variations in partial specific volumes of lipids and proteins, adopted from the literature, result in negligible differences in the calculated Lp-P compared with experimental measurement variations in concentration and size (Figure S1 C, D). (3) The total molecular volume of the main apos (A1, A2, C1, C2, C3, B, and E) and lipid classes (FC, CE, TG, PL) accounts for the total Lp particle volume in each fraction, with negligible contributions from other small proteins or from larger proteins present only in minor particle subpopulations. (4) HDL and LDL particles are assumed to be surrounded by two theoretical water layers; thus, all measured hydrodynamic diameters (d_DLS_) were subtracted by 1.2 nm to estimate d_m_ in Eqs. 5 and 6.

### Definition of HDL and LDL subclasses based on composition differences

**Fig. 2:**
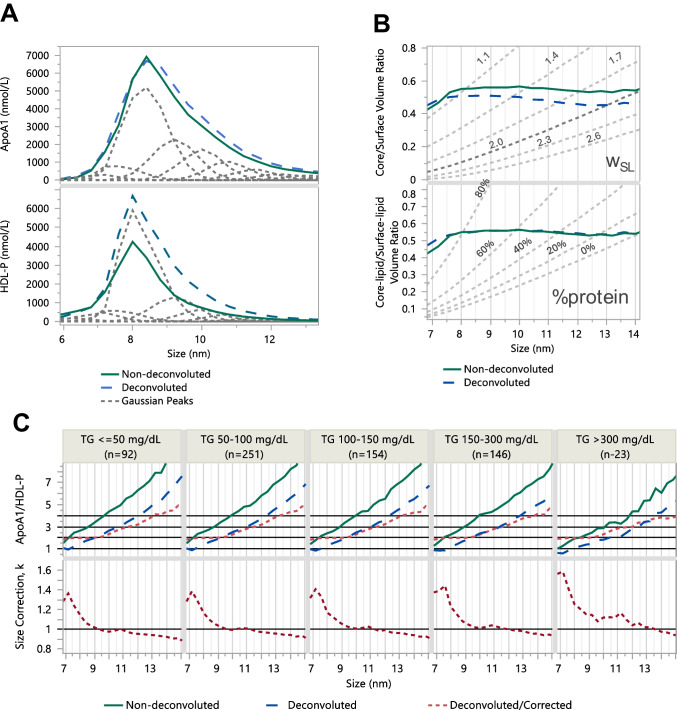
Comparison of average non-deconvoluted (solid line) and deconvoluted HDL data (dashed line). **A** Average apoA1 profile (top) and HDLP profile (bottom) with overlaid average of deconvoluted Gaussian subspecies (dotted line). **B** Estimation of the surface layer thickness (w_SL_) and % protein volume in a 2 nm monolayer, based on intercept of volume ratios with geometrically constrained expectations (gray dotted lines). **C** ApoA1/HDL-P (top) stratified by total TG concentrations. Dotted line shows ApoA1/HDL-P after correction of d_m_ for discoidal shape HDL (top), using corresponding *k* corrections (bottom). To evaluate average concentration profiles, the size scale of the AF4 profiles was binned into 0.75, 1.5, and 3 nm increments in the HDL, LDL, and VLDL regions, respectively (1–2 fractions per bin) and the fraction concentrations were summed within each size bin. Note: Deconvolution resulted in higher HDL-P and less overestimation of apoA1/HDL-P, still, achieving ~ 2 apoA1/HDL-P for small HDL of 7.5–8 nm also required correction of d_m_ with *k* of 1.2–1.3

**Fig. 3:**
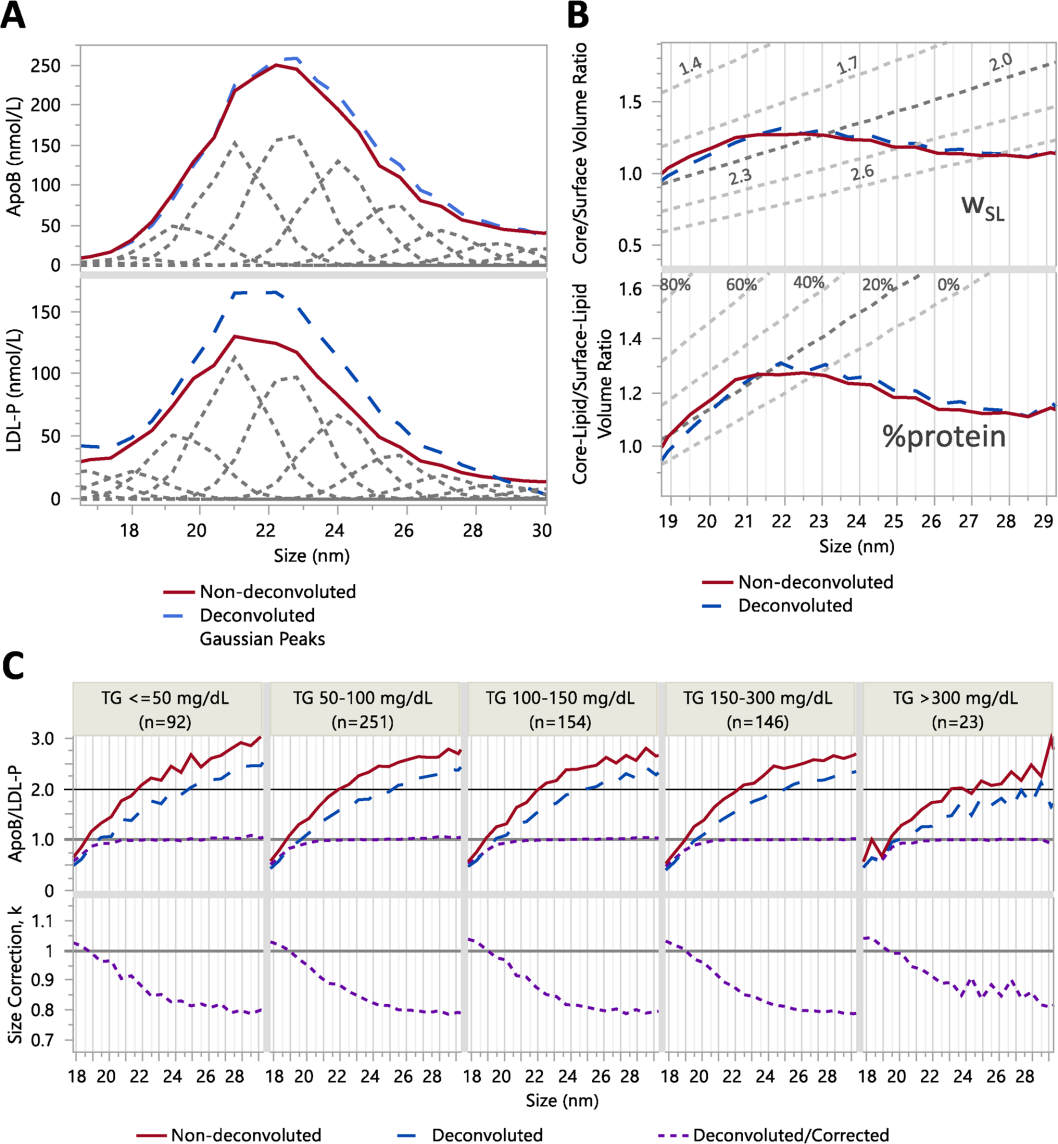
Comparison of average non-deconvoluted (solid line) and deconvoluted LDL data (dashed line). **A** Average apoB profile (top) and LDL-P profile (bottom) with overlaid average of deconvoluted Gaussian subspecies (dotted line). **B** Estimation of the surface layer thickness (w_SL_) and % protein volume in a 2 nm monolayer, based on intercept of volume ratios with geometrically constrained expectations (gray dotted lines). **C** ApoB/HDL-P (top) stratified by total TG concentrations. Dotted line shows ApoB/HDL-P after correction of d_m_ for LDL dimerization using corresponding *k* corrections (bottom). To evaluate average concentration profiles, the size scale of the AF4 profiles was binned into 0.75, 1.5, and 3 nm increments in the HDL, LDL, and VLDL regions, respectively (1–2 fractions per bin) and the fraction concentrations were summed within each size bin. Note: Deconvolution resulted in higher LDL-P and less overestimation of apoB/LDL-P, still achieving ~ 1 apoB/LDL-P for medium and large LDL of 22–28 nm also required correction of d_m_ with *k* of 0.9–0.8

Total concentrations of the main proteins and lipids were summarized in Table S3. The presence of HDL and LDL subspecies with distinct protein compositions was supported by visible variations in the shapes of size profiles, differences between protein and lipid profiles within samples (Figure S1C), and by differences between samples in the size profiles of specific lipids and proteins (Figure S1D). Although all samples were fractionated individually, this study primarily assessed the average size distribution of proteins and lipids across all samples or within sample groups stratified by total-TG concentration ranges (Figs. 2 and 3 and Figures S2–S7). Although we originally expected a strong correlation of HDL and LDL size distributions with CAD outcome in the CATHGEN cohort, especially the correlation of LDL size with CAD diagnosis or CAD score (based on number/severity of blockages). However, we found no significant differences by CAD.

Although the precise number and sizes of the distinct HDL and LDL subspecies were unknown, we deconvoluted the experimental profiles for each analyte and each sample, assuming a fixed number of theoretical subspecies distributed at equal size increments: 0.75 nm for HDL and 1.5 nm for LDL. The percent accuracy of the deconvoluted data relative to the experimental data was calculated by dividing the sum of Gaussian subspecies concentrations by the sum of the measured concentrations, for each analyte and size increment within the HDL and LDL size ranges, as summarized in supplemental Tables S4–S7. Each subspecies was modeled with a set of Gaussian peaks of equal half-peak width. The chosen increment and peak width of the individual Gaussian peaks reflect the resolution capability of our AF4 separation method, as demonstrated in our previous study [28].

**Fig. 4:**
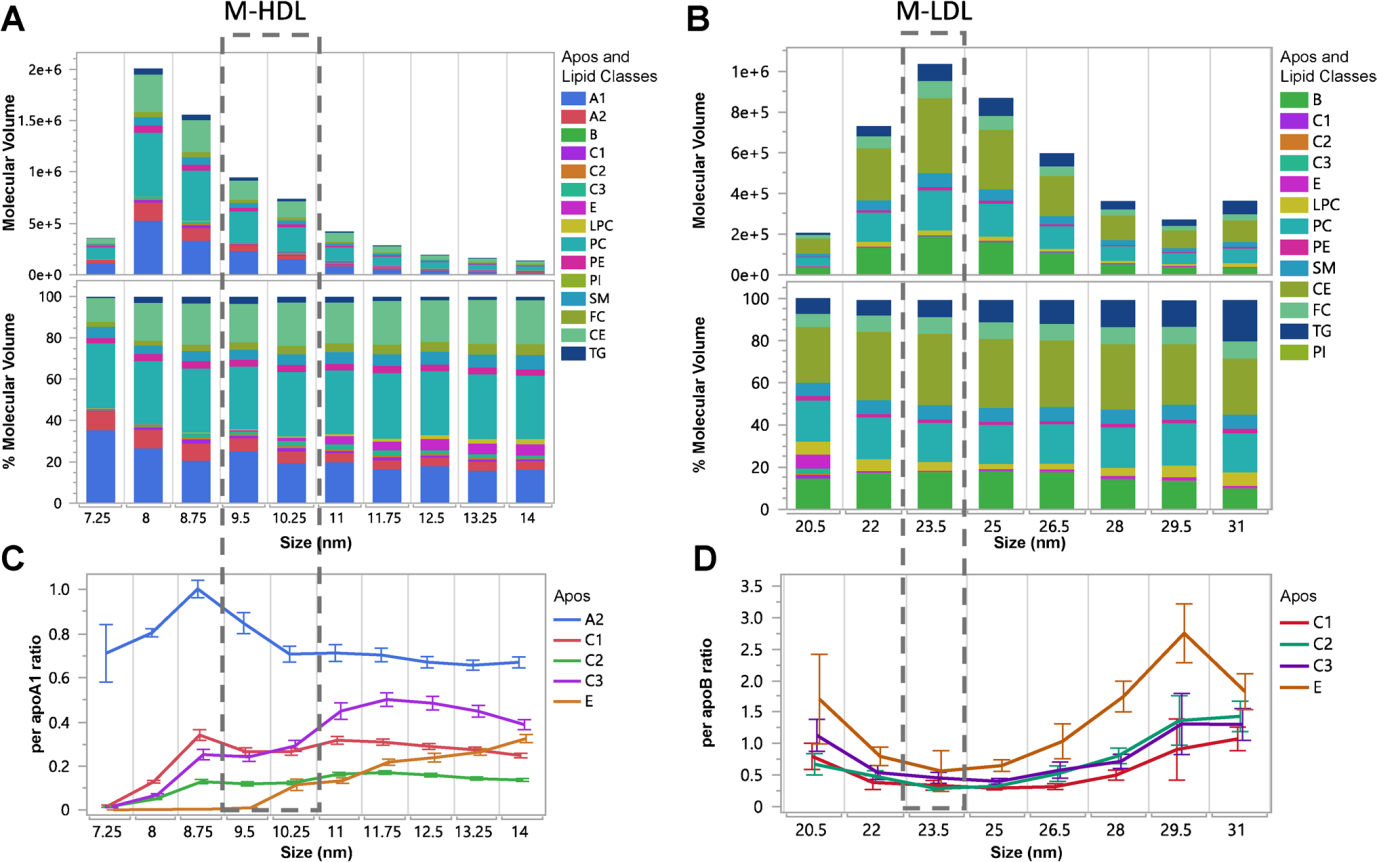
Absolute molecular volume and relative %volume contributions by main proteins and lipids to HDL particle volume (**A**), and LDL particle volume (**B**). Average number of apoA2, apoC1, apoC2, apoC3, and apoE per apoA1 for HDL (**C**), and average number of apoC1, apoC2, apoC3, and apoE per apoB for LDL (**D**). Error bars indicate 5th–95th confidence intervals (for better visibility, standard deviation bars were about 3–5 times wider). HDL subclasses: S-HDL (7.25 and 8.75 nm), M-HDL (9.5 and 10.25 nm), and large HDL (L-HDL) (11–14 nm). LDL subclasses: S-LDL (20.5 and 22 nm), M-LDL (23.5 nm), and L-LDL (25–31 nm). Note: Calculated ratios/apoA1 and/apoB < 1 indicate that not all particles carry that apolipoprotein. Overlap of S-LDL with apoE-containing particles without apoB caused higher/apoB ratios for 20.5 and 22 nm LDL (**D**)

The deconvoluted nmol/L concentrations, calculated using Eq. 4, were converted to absolute molecular volumes and % molecular volume contributions within each subspecies, depicted as stacked bar graphs, averaged across all samples in Fig. 4, and stratified by total TG concentrations in Figure S8.

The trends in % molecular volume abundances were primarily driven by increased proportions of TG, at 9.5–10.25 nm for HDL and around 23.5 nm for LDL (Tables 1 and 2). The % molecular volume of TG was also associated with changes in the concentration ratios of apos A2, C1, C2, C3, and E relative to apoA1 for HDL and apoB for LDL (line graphs in Fig. 4 and Figure S8). The HDL and LDL size ranges near the identified inflection points were designated as medium-size subclasses. The 9.5 nm and 10.25 nm subspecies were classified as medium HDL (M-HDL), and the 23.5 nm subspecies as medium LDL (M-LDL), with subspecies of smaller and larger sizes classified as small and large, respectively.

**Table 1:** Percent volume contribution of apolipoproteins and lipid classes in deconvoluted HDL subspecies (n = 666)

**Subclass**	**S-HDL**			**M-HDL**		**L-HDL**				
**Subspecies (nm)**	**7.25**	**8**	**8.75**	**9.5**	**10.25**	**11**	**11.75**	**12.5**	**13.25**	**14**
%apoA1	24 (23)	28 (6)	22 (5)	23 (6)	22 (6)	19 (6)	17 (6)	16 (5)	16 (5)	15 (4)
%apoA2	7 (13)	9 (3)	9 (3)	8 (5)	6 (3)	5 (3)	5 (2)	4 (2)	4 (2)	4 (2)
%apoC1	0.3 (1.5)	1 (1)	2 (1)	2 (1)	1 (1)	2 (1)	1 (1)	1 (1)	1 (1)	1 (1)
%apoC2	0.3 (1.3)	1 (1)	1 (1)	1 (1)	1 (1)	1 (1)	1 (1)	1 (1)	1 (1)	1 (1)
%apoC3	0.3 (2.7)	1 (1)	2 (2)	2 (1)	2 (1)	2 (2)	2 (1)	2 (1)	2 (1)	2 (1)
%apoE	1 (6)	1 (5)	0.4 (3.3)	1 (3)	3 (7)	3 (4)	5 (4)	5 (4)	5 (4)	6 (4)
%LPC	6 (13)^a^	0.3 (3.4)^a^	0.3 (0.7)	0.4 (0.5)	1 (2)	1 (1)	1 (1)	1 (1)	2 (1)	2 (2)
%PC	36 (27)	30 (6)	31 (6)	29 (7)	30 (7)	30 (8)	30 (7)	30 (7)	29 (7)	28 (7)
%PE	2 (4)	3 (2)	4 (2)	3 (2)	3 (2)	3 (2)	3 (2)	3 (2)	3 (2)	3 (1)
%SM	8 (12)	4 (1)	4 (1)	5 (2)	5 (2)	5 (2)	6 (2)	5 (2)	6 (2)	6 (2)
%FC	2 (5)	2 (1)	3 (1)	3 (1)	3 (1)	4 (1)	4 (1)	4 (1)	4 (1)	4 (1)
%CE	2 (5)	18 (5)	19 (5)	19 (5)	19 (5)	20 (6)	19 (6)	20 (5)	19 (5)	19 (6)
%TG	12 (22)	3 (2)	3 (2)	5 (3)	3 (4)	5 (4)	7 (4)	7 (4)	8 (4)	12 (5)

^a^%LPC contribution is bound mainly to albumin and not included in the calculation of HDL-P

**Table 2:** Percent volume contribution of apolipoproteins and lipid classes in deconvoluted LDL subspecies (n = 666)

**Subclasses**	**S-LDL**			**M-LDL**	**L-LDL**				**VLDL**
**Subspecies (nm)**	**19**	**20.5**	**22**	**23.5**	**25**	**26.5**	**28**	**29.5**	**32.5**
apoB	9 (10)	15 (14)	17 (7)	18 (6)	18 (5)	18 (6)	14 (6)	13 (10)	13 (14)
apoC1	1 (2)	2 (5)	0.1 (0.1)	0.1 (0.2)	0.1 (0)	0.1 (0)	0.1 (0.1)	0.1 (0.1)	0.3 (0.3)
apoC2	1 (1)	1 (2)	0.1 (0.2)	0.1 (0.2)	0.1 (0.2)	0.1 (0.2)	0.2 (0.3)	0.2 (0.3)	0.2 (0.4)
apoC3	1 (2)	2 (6)	0.1 (0.2)	0.2 (0.9)	0.1 (0.2)	0.1 (0.2)	0.2 (0.3)	0.2 (0.3)	0.4 (1.1)
apoE	6 (10)^a^	7 (15)^a^	1 (1)	0.5 (0.9)	1 (1)	1 (1)	1 (2)	2 (2)	3 (5)
LPC	3 (6)	6 (9)	6 (9)	4 (10)	2 (3)	3 (6)	4 (3)	6 (8)	13 (24)
PC	26 (14)	20 (15)	20 (6)	18 (5)	18 (4)	18 (5)	19 (7)	20 (9)	15 (12)
PE	3 (3)	2 (4)	2 (1)	1 (1)	1 (1)	1 (1)	2 (1)	2 (1)	1 (2)
SM	7 (5)	6 (6)	7 (2)	7 (4)	6 (2)	7 (2)	7 (3)	7 (5)	6 (8)
CE	24 (16)	26 (18)	32 (9)	34 (9)	33 (7)	32 (8)	31 (9)	29 (12)	18 (13)
FC	7 (5)	6 (6)	8 (2)	8 (2)	8 (2)	8 (2)	8 (2)	8 (4)	6 (5)
TG	12 (11)	7 (11)	7 (4)	8 (4)	11 (5)	11 (6)	13 (8)	12 (8)	23 (19)

^a^%apoE i contribution is bound mainly to apoE-only particles and not included in the LDL-P calculation

### Effect of deconvolution on calculation of Lp-P count

For individual lipid classes and proteins, we achieved a good match between the non-deconvoluted and the summed deconvoluted Gaussian subspecies, as shown for apoA1 in Figs. 2A and apoB in Fig. 3A. Average overlays of non-deconvoluted and deconvoluted profiles stratified by TG ranges are compared in Figures S2–S7. 

Lp-P was calculated for each AF4 fraction and for Gaussian subspecies using equations shown in supplemental information. Substantially different Lp-P profiles emerged when d_m_ was derived from the fraction-based d_DLS_ measurements or when d_m_ was based on the peak maxima of the Gaussian subspecies (Figs. 2A and 3A, bottom graphs). Without deconvolution, the calculated Lp-P across fractions was systematically underestimated. The explanation lies in the underlying mathematical formulas used for the two calculations, as illustrated by the simulation in Figure S11. On average, treating size as a continuous fraction-to-fraction variable, as opposed to a quantized subspecies-to-subspecies variable, causes 20–40% underestimation of the HDL-P and LDL-P. Underestimation of Lp-P also caused overestimation of apoA1/HDL-P and apoB/LDL-P ratios, as shown by the overlays in Figs. 2C and 3C, highlighting the importance of profile deconvolution.

### Differences between subspecies of HDL and LDL in surface layer thickness and %protein volume in the monolayer

As implied by our conceptual models in Fig. 1, with a constant surface layer thickness (w_SL_), the core/surface volume ratio is geometrically constrained by particle size. Similarly, with a constant %protein volume in a 2 nm monolayer, the core-lipid/surface-lipid volume ratio is also constrained. Core/surface volume ratios were calculated for w_SL_ of 1.4, 1.7, 2.0, 2.3, and 2.6 nm, and core-lipid/surface-lipid ratios were calculated for 80%, 60%, 40%, 20%, and <1% protein proportions. These theoretical isobar contours are depicted as gray dotted lines in Fig. 2B and Figure S12 for HDL subspecies, and Fig. 3B and Figure S13 for LDL subspecies. By overlaying the experimentally derived ratio-versus-size profiles with these theoretical contours, the intersections allowed estimation of the w_SL_ and the %protein in the monolayer that most closely aligned with the experimental data.

For HDL, the estimated w_SL_ varied from 1.1 to 1.4 nm for S-HDL and from 1.6 to 1.8 nm for L-HDL (Fig. 2A). The corresponding %protein volume in the monolayer was 80% to 60% for S-HDL and 60% to 40% for L-HDL (Fig. 2B). For S-HDL, we surmised that the apparent contradiction between a thin surface layer (w_SL_ < 1.8 nm) and high protein volume in the monolayer (%protein > 60%) was most consistent with the discoidal HDL model. In the LDL size range, w_SL_ varied from 1.8 nm for S-LDL to 2.6 nm for L-LDL. The corresponding %protein volume in the monolayer was ~ 30% for S-LDL and < 0% for L-LDL (Fig. 3B). A %protein of < 0% indicates that, even without any protein contribution to the monolayer, the surface-lipid volume exceeds that needed to cover the core. We infer that the apparently thick surface, lack of protein volume in the monolayer (%protein < 1%), and excess of surface-lipid volume can be explained by 17–20 nm S-LDL particles in the fractions being misclassified as L-LDL, most likely due to the formation of S-LDL dimers.

### ApoA1/HDL-P and apoB/LDL-P calculation

The accuracy of the apoA1/HDL-P and apoB/LDL-P ratios was assessed by comparing them to the general consensus reported across numerous literature studies, which report 2, 3, and 4 apoA1 molecules per HDL particle, and 1 apoB molecule per LDL particle. Even with deconvolution, which should have minimized estimation bias, we found lower-than-expected values, with 1–1.5 apoA1/HDL instead of 2 for S-HDL at 7–8 nm (Fig. 2), and higher-than-expected values, with 1.5–2 apoB/LDL-P for L-LDL at 24–26 nm (Fig. 3). For S-HDL, the underestimation of apoA1/HDL-P aligned with estimated w_SL_ of 1.2–1.5 nm and %protein of 70–80. For L-LDL, the overestimation of apoB/LDL-P corresponded to w_SL_ values greater than 2 nm and protein volume proportions of less than 1% in the monolayer. Therefore, we conclude that the lower-than-expected apoA1/HDL-P and higher-than-expected apoB/LDL-P values were likely influenced by the presence of discoidal HDL and LDL dimers.

### Correction to apoA1/HDL-P and apoB/LDL-P target values consistent with presence of HDL disks and LDL dimers

Based on the alternative conceptual models depicted in Fig. 1B, we inferred that HDL disk and LDL dimer formation introduce substantial disproportionality between the measured hydrodynamic diameter (d_DLS_) and the mass diameter (d_m_), necessitating a correction factor k (Eq. 6). We “back-calculated” the k-factors using Eq. 7 for HDL and Eq. 8 for LDL subspecies.

For HDL particles sized 7.5–8 nm, achieving ~ 2 apoA1/HDL-P required 1.7–2.1 times decrease in HDL-P resulting in a* k* factor of 1.2–1.3 (Fig. 2C). For LDL particles sized 22–26 nm, achieving 1 apoB/LDL-P necessitated 2–3 times increase in LDL-P, resulting in a *k* factor of 0.7–0.8 (Fig. 3C). In other words, our d_DLS_ measurements underestimated d_m_ for S-HDL and overestimated d_m_ for M-LDL and L-LDL. For S-HDL, k = 1.2–1.3 was consistent with discoidal shape. For M/L-LDL, k = 0.7–0.8 matched our geometric predictions for LDL dimers.

### Comparison of Lp-P via AF4-LC-MS/MS and NMR platforms

We obtained apoA1, apoB, HDL-P, and LDL-P data from NMR analysis, enabling direct comparison between AF4-LC-MS (MS in short) and NMR platforms. Based on total apoA1 concentrations, the MS and NMR platforms showed a correlation with an R^2^ = 0.63 (slope = 0.86) and a mean percent difference of 3.3% (MS-NMR)/NMR (Figure S14A); whereas total apoB concentrations correlated with an R^2^ of 0.68 (slope = 1.03) and a mean difference of 22% (Figure S14B and Figure S15B).

**Fig. 5:**
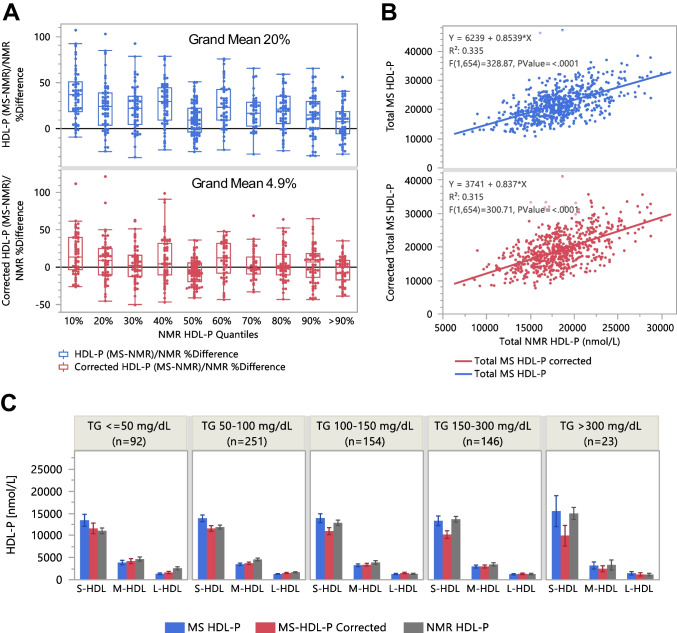
Comparison of AF4-LC-MS/MS and NMR derived HDL-P. **A** Differences of the uncorrected and corrected total HDL-P by AF4-LC-MS/MS relative to NMR HDL-P across NMR HDL-P quantiles. **B** Correlation of uncorrected and corrected total HDL-P by AF4-LC-MS/MS versus NMR HDL-P. **C** Comparison of sums of Gaussian subspecies by S-HDL (7.25–8.75 nm), M-HDL (9.5–10.25 nm), and L-HDL (11–14 nm) with NMR data. Note: Total HDL-P % differences were less after correction based on apoA1/HDL-P, consistent with discoidal S-HDL

**Fig. 6:**
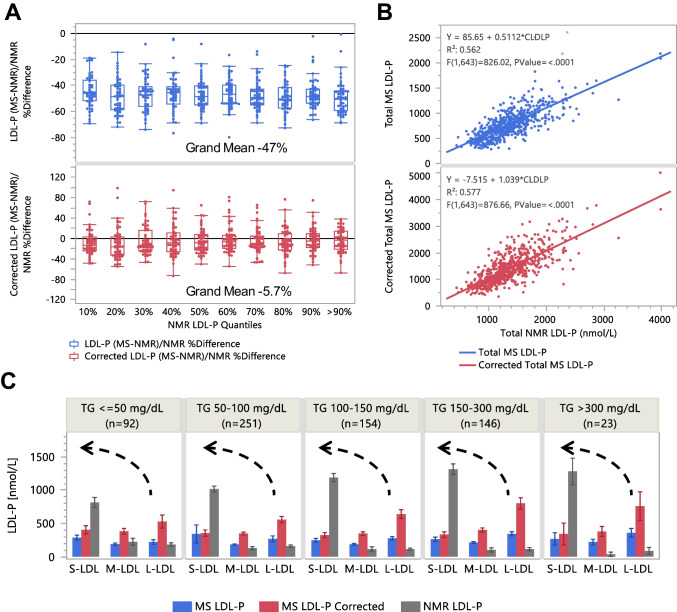
Comparison of AF4-LC-MS/MS and NMR derived LDL-P. **A** Differences of the uncorrected and corrected total LDL-P by AF4-LC-MS/MS relative to NMR LDL-P across NMR LDL-P quantiles. **B** Correlation of uncorrected and corrected total LDL-P by AF4-LC-MS/MS versus NMR LDL-P. **C** Comparison of sums of gaussian subspecies by S-LDL (19–22 nm), M-LDL (23.5 nm), and L-LDL (25–29.5 nm) with NMR data**.** Note: Total LDL-P % differences were less after correction based on apoB/LDL-P, consistent with dimerization of S-LDL. The k-correction doubled the number of LDL-P in the L-LDL size range (**C**, red bars). The dashed arrows imply that re-grouping these particles as S-LDL would result in S-LDL being the most abundant subclass, with closer agreement with the NMR results

Without k correction, differences relative to the NMR data for total HDL-P and total LDL-P (sums of 10 and 8 subspecies, respectively) were 20% and −47%, respectively (Figs. 5A and 6A). After k correction, these differences decreased to 4.9% and −5.7%, respectively. The x-y correlation R^2^ values remained similar before and after k correction. The MS-NMR correlation slope remained approximately 0.8 for total HDL-P but increased from approximately 0.5 to approximately 1.0 for LDL-P (Figs. 5B and 6B). These results demonstrate that adjusting HDL-P and LDL-P of subclasses based on consensus apoA1/HDL-P and apoB/LDL-P values can bring orthogonal analytical platforms into closer agreement.

On average, the relative HDL-P across subclasses exhibited similar trends on both platforms, identifying S-HDL as the predominant subclass (Fig. 5C). However, greater discrepancies were observed for the LDL subclasses. The k-correction approximately doubled the LDL-P in the L-LDL size range (Fig. 6C, red bars). The dashed arrows indicate that re-grouping these particles as S-LDL would result in S-LDL being the most abundant subclass, resulting in a closer agreement with the NMR results. The necessity for reclassification provides further evidence that a significant percentage of the S-LDL particles were present as S-LDL dimers, which appeared in the M-LDL and L-LDL size ranges.

## Discussion

### Overview

The aim of this study was to explore systematic biases in calculating lipoprotein particle numbers (Lp-P) based on particle volume and molecular volume assumptions. We followed a series of novel, key steps, including high-resolution separation of lipoproteins into narrow size fractions, detailed quantification of lipoprotein constituents, and deconvolution of concentration-size profiles into Gaussian subspecies. We constructed particle models that predicted experimentally measurable structural features, most notably, deviations from spherical geometry. These predictions were also evaluated based on the independently derived shape-correction factors (k) used to adjust HDL and LDL mass diameters (k*d_m_), from apoA1/HDL-P and apoB/LDL-P with established consensus values, which provided an independent estimate of the required k-factors. After correction, Lp-P values aligned closely with those obtained by NMR for the same samples, including the dominant presence of S-HDL and S-LDL subclasses, demonstrating that particle shape plays a critical role in volumetric Lp-P calculations. Our findings underscore the importance of correcting total and subclass-specific Lp-P measurements based on apoA1/HDL-P and apoB/LDL-P consensus values to reconcile discrepancies across platforms due to the presence of non-spherical particles such as s-HDL disks and S-LDL dimers.

### Nuts and bolts of the volumetric approach

Ideally, Lp-P calculations should be based on size and concentration measurements obtained from structurally homogeneous particle isolates. However, no single physical property of lipoproteins allows resolution of all lipoprotein subspecies in plasma. Two- or three-dimensional methods have therefore been employed, where size separation is coupled with other orthogonal techniques, based on electrostatic charge, density, immuno-affinity, or precipitation propensity. These multi-dimensional methods involve several buffer exchange steps and subject the Lp particles to various shearing forces. Consequently, every separation approach requires a trade-off between structural integrity and purity.

In our study, choosing AF4 as a one-dimensional, gentle size separation approach enabled the preservation of structural integrity while providing superior size resolution. AF4 allowed the resolution of HDL and LDL particles with ± 0.7 nm half-peak width and ± 1.5 nm half-peak width, coupled with fraction collection in 0.3–0.7 nm and 0.7–1.5 nm increments, respectively. Even with this enhanced resolution, we were not able to obtain compositionally homogenous isolates. Each fraction therefore only reflected the average properties of multiple subspecies. We addressed this inherent lack of resolution by collecting fractions in narrow size increments and performing precise size and concentration measurements in each fraction. The HDL and LDL profiles were distributed in 22 and 12 fractions, providing enough experimental points to be able to model each size profile by deconvolution into Gaussian subspecies.

The accuracy of the size measurements was critical to all our calculations. All measurable size-sensitive properties affected by measurement conditions (e.g., ionic strength, viscosity, and pH), as well as particle properties (i.e., purity, polydispersity, shape, charge, and aggregation propensity) [42–44]. Even in electron microscopy (EM), the apparent size of a nanoparticle depends on the projected area, edge contrast, and staining parameters [42]. Consequently, on the nm-scale, no size measurement technique can be considered inherently correct [45, 46]. We chose DLS as a size measurement technique. Compared to other techniques, hydrodynamic size measurement by DLS is non-destructive, precise and does not require external calibration. Coupling AF4 and DLS was advantageous because AF4 retention times have a theoretically established direct relationship with the hydrodynamic diameter of particles, which is similarly affected by matrix parameters as the hydrodynamic diameter measured by DLS. This similarity of AF4 and DLS provided sound justification for the empirical conversion of our concentration profiles from fraction number scale to hydrodynamic size scale.

After conversion from concentration-fraction number to concentration-size profiles, we performed profile deconvolution into 10 HDL and 8 LDL Gaussian subspecies. Calculating Lp-P with and without deconvolution highlighted an important source of bias arising from the use of size as a continuous fraction-based variable rather than as a discrete characteristic of HDL and LDL subspecies. Comparison of Lp-P without versus with deconvolution showed that overlap of particles with relatively smaller particles (which contribute proportionately less molecular volume for the expected size) results in underestimation of their Lp-P, whereas overlap of particles with larger particles (which contribute proportionately more molecular volume for the expected size) leads to overestimation of their Lp-P. The degree of these under- and overestimations of Lp-P depends on the relative abundance of the overlapping smaller and larger subspecies. At a similar molecular volume contribution, the underestimation due to overlap with smaller subspecies may cancel out the overestimation due to overlap with larger subspecies, most likely near the size profile maximum. However, the lack of deconvolution can be a significant source of bias on both the ascending and descending sides of the size profiles. For these reasons, deconvolution of the continuous HDL and LDL size profiles was a key step toward more accurately calculating Lp-P and per-particle molecular stoichiometries.

Beyond accurate size and concentration measurements and deconvolution, estimation of particle mass diameters (d_m_) was essential. The mass diameter represents the diameter of a sphere with a volume equal to the sum of the molecular volumes of all particle constituents that cannot be measured directly. For each fraction, d_m_ had to be inferred from the hydrodynamic diameter which is affected by particle geometry (Fig. 1 and Figure S10). Our main evidence for deviations from spherical particle shape was derived from structural indicators, including surface layer thickness (w_SL_), % protein volume in the monolayer, and apoA1/HDL and apoB/LDL estimates. Our reference was spherical shape, with expected w_SL_ ~ 2 nm, a % protein volume of 20–40% [47], 2–4 apoA1/HDL-P, and 1 apoB/LDL, based on well-established literature consensus, verified by cross-linking, molecular weight measurements [48–50] and microscopic imaging [51, 52].

For M/L-HDL, our graphical estimates aligned with expected values for spherical particles. For S-HDL, however, we estimated w_SL_ < 1.5 nm, %protein > 60, and apoA1/HDL of 1–1.5. The apparently thin surface could be explained by the lack of monolayer-core phase separation. The high %protein was consistent with a torus-like apoA1 scaffold surrounding the randomly packed lipids. We therefore surmised that the discoidal shape of S-HDL particles results in a hydrodynamic radius that is smaller than the radius of rotation and the radius of gyration (Figure S10), leading to an underestimation of the mass radius for discoidal S-HDL particles, requiring correction with k > 1.

For S-LDL, our w_SL_ and % protein estimates were consistent with a standard spherical LDL model. For L-LDL, however, we estimated not only a thicker surface (w_SL_ = 2.3–2.6 nm), but also estimated % protein < 0%, suggesting no embedding of protein domains and excess surface-lipid volume covering a disproportionately small core. In addition, apoB/LDL of 1.5–2 were calculated. The same core/surface and core-lipid/surface-lipid ratios yielded normal range estimates (w_SL_ ~ 2 nm, % protein ~ 20, apoB/LDL ~ 1) if the particles were 18–21 nm instead of 25–28 nm. Therefore, we concluded that a substantial number of S-LDL particles in these L-LDL size fractions most likely form dimers. In terms of particle shape, these dimers must be cylinder-like, and the hydrodynamic radius must be smaller than a radius of rotation, but larger than the hydrodynamic radius of the monomers inside the dimer (cylinder), requiring k-correction < 1.

### Protein composition differences corresponding with HDL disk and LDL dimer presence

We included apos A2, C1, C2, C3, and apoE as contributors to the particle volume. However, these apos, with the exception of apoE, have molecular weights of ~ 10 kDa that contribute minimally to the total molecular volume in the fractions compared with lipids plus apoA1 or lipids plus apoB, resulting in a negligible impact on HDL-P and LDL-P estimates. ApoE, with a molecular weight of 34 kDa, is larger than apoA1 (28 kDa) but much smaller than apoB (550 kDa). In L-HDL fractions, the apoE/apoA1 ratio was < 0.2, and in LDL fractions the ApoE/apoB ratio was up to 4, indicating that apoE’s volume contribution was small relative to L-HDL or L-LDL particle volume.

Differences in protein composition reflect variations in surface properties and structure between small and large subclasses. For example, discoidal S-HDL was distinguished from L-HDL (HDL2) by a higher apoA2/apoA1 ratio, whereas spherical L-HDL exhibited higher apoC2/apoA1 and apoC3/apoA1 ratios. Given the high % protein volume in S-HDL, we hypothesize that apoA2 and apoCs bind primarily to the surface of S-HDL and may embed more extensively into the monolayer in L-HDL. 

Additionally, lower total TG levels were associated with greater overestimation of apoB/LDL-P, indicating increased LDL dimerization. Exchangeable apolipoproteins/apoB ratios < 0.2 at ~ 20 nm suggested reduced binding compared to LDL particles > 24 nm (Fig. 4B). We observed that at lower versus higher TG levels, there were fewer apoCs/apoB but more apoE/apoB (Figure S8B). These trends suggest that reduced apoC and increased apoE binding to LDL may promote dimerization, which in turn facilitates interaction with the LDL receptor. We further hypothesize that ApoE-enriched S-LDL particles are more likely to dimerize and appear as L-LDL. Because ApoE mediates binding to the LDL receptor (LDLR), apoE-containing dimerized S-LDL particles are expected to have shorter circulation times. In contrast, the non-dimerized S-LDL particles with lower apoE and higher apoC3 content exhibit reduced LDLR affinity, consistent with the longer circulation time of S-LDL particles [53].

Notably, in our earlier study comparing normolipidemic and hyperlipidemic individuals, we observed a clear association between high total TG levels and higher PE/SM and lower apoE/apoC3 ratios on M-/L-LDL, suggesting a connection between TG-related surface fluidity and competitive binding of apoC3 and apoE [28]. Collectively, these hyperlipidemia-related confounding effects, together with potential dimerization, may explain the elusive nature of s-LDL as a biomarker for atherosclerosis risk when defined solely by measured size, as recognized in recent clinical guidelines [54].

Previous reports using apoC3 immunoprecipitation followed by ultracentrifugation showed low ratios of apoE/apoC3 ≈ 0.09–0.15 on apoC3-containing LDL isolates, whereas prior to immune isolation they reported ratios of apoE/apoB ≈ 1.3–2.4, in the range of our LDL fractions. Two effects contribute to the apoE/apoC3 ratio: (1) ApoC3 and ApoE are distributed differently in LDL size-fractions (Figure S17). The average apoE profile maximum occurred at a ~ 2 nm larger size (22 nm vs. 24 nm) compared with apoCs and apoB, providing evidence that apoE is primarily associated with M/L-LDL particles. This compositional difference was enhanced by calculation of per apoB ratios from the deconvoluted data (Fig. 3D). (2) By design, the apoC3-affinity and UC-based isolation of LDL particles enrich apoC3-containing LDL with reduced apoE content, thereby increasing the apoC3/apoB ratio and decreasing the apoE/apoB ratio. This is consistent with our observation that the profile maxima for apoE was at larger size than for apoCs and apoB. Therefore, apoE/apoC3 ratios reported by Mendivil et al. support our conclusion that apoE-containing LDL contains less apoC3 and multiple copies of apoE/apoB.

The exact percentage of L-LDL-sized particles that represent dimers cannot be determined from our data. We can only infer their presence based on the apoE/apoC3, apoE/apoB, and apoC3/apoB ratios. Nonetheless, we propose that further investigation of the apparent association of LDL dimerization and the constellation of LDL-carried lipids, apoE, and apoC3 may be critical for better understanding extended circulation times and oxidative modification of LDL particles that more likely contribute to atherosclerosis development [16–18]. Our working hypothesis is that the apoE-mediated s-LDL dimers may be less atherogenic because they are more efficiently cleared by LDL receptors (LDLR). Disruption of s-LDL dimers by apoC3, in contrast, may extend circulation, thereby increasing opportunities for lipid oxidation and promoting formation of more atherogenic s-LDL. Additionally, mechanisms of LDL dimerization may also exist, such as interactions with circulating cleaved domains of LDLR. Notably, recent studies found that LDLR may be co-expressed and co-internalized as dimers [52, 55] and able to form 1:2 and 2:2 LDL-LDLR complexes [52].

### Other literature evidence of structural alterations of LDL

We are not the first to show evidence of greater-than-expected apoB/lipid mass ratios [27, 56]. McNamara et al. suggested that apoB-100 occupies a larger surface volume on L-LDL particles, resulting in less embedding of apoB into the monolayer, leading to a “thicker” surface and smaller core diameter [27]. We considered such alternative models in Fig. 1B and estimated a thicker surface, w_SL_ > 2 nm, which would decrease the core diameter. Although we estimated w_SL_ = 2.3–2.6 nm, we surmised that a w_SL_ increase of only 0.3–0.6 nm would result in a 0.6–1.2 nm decrease in core diameter, which is insufficient to explain the observed core-lipid/surface-lipid ratio derived from our data. Expansion of a single apoB into the hydrated protein layer by ~ 2 nm (w_SL_ ~ 4 nm) and a corresponding core diameter decrease ~ 4 nm would be necessary. A monomeric LDL particle with a thicker surface and a single apoB is feasible only if it is substantially smaller than we measured. Using our measured LDL size, we calculated 2–3 apoB/LDL-P values (based on spherical geometry). In other words, the necessarily small monomers would need to be contained within a larger cylindrical shaped dimer that exhibits both a thick surface and more than 1 apoB per particle.

Teerlink et al. proposed discoidal shaped LDL particles, with apoB circling the particle twice, to explain their greater than expected apoB/lipid mass ratios and larger measured diameters than those estimated from lipid/apoB mass ratios [56]. Flattened LDL shape was also shown by using Small Angel X-ray Scattering (SAXS) [57], as a result of lamellar organization of CE molecules inside the LDL core, more likely occurring after Cu^2+^ induced oxidation [58, 59]. The distribution of the imbedded apoB structure may contribute further distortion to the discoidal shape as suggested by Teerlink et al. A flattened or discoidal LDL shape with 0.5*V_m_ (~ 0.5d_in_*d_m_^2^) would lower the calculated apoB/LDL-P ratio from 2 to 1. However, we surmise that a quasi-discoidal LDL shape would also result in smaller measured d_DLS_ and elution in the smaller LDL size range. Additionally, the Teerlink model would result in higher core-lipid/surface-lipid ratio, inconsistence with lower core-lipid/surface-lipid ratio measured in our l-LDL fractions. Therefore, we assert that whether discoidal or spherical, S-LDL particles must dimerize to be observed in the L-LDL size range.

Evidence of LDL dimerization has been reported in other studies [16–18], where dimerization was induced by heating, vortexing, oxidation, and sphingomyelinase lipolysis [18, 60, 61]. Dimerization and fusion were more likely with increased glycosylation [17]. Sphingomyelinase-induced LDL aggregation in human samples was also influenced by diet and ethnicity [18, 61]. Intriguingly, we found no prior reports of spontaneous LDL dimerization using other sizing techniques, whereas we observed it with AF4. We speculate that during AF4 separation and dilution, all particles move near the membrane-covered wall in a slow-moving laminar layer, approximately 10% of the channel thickness (0.1*0.5 mm). Due to applied flow splitting at the channel outlet, particles are diluted relative to plasma by only 5–20-fold immediately before fraction collection. This feature distinguishes AF4 separation from other separation techniques. In our previous study optimizing AF4 lipoprotein size separation [29], we diluted plasma with delipidated plasma and injected 80 µL containing 0–100% of the native sample. The lack of shift in the LDL size profile and similar apoB recovery indicated no significant aggregation or membrane binding due to increasing concentration of LDL particles. In this pilot study, injecting 80 µL from 150 serum samples, we also calculated 1–3 apoB/LDL-P but without deconvolution [28]. Nonetheless, as a precaution, in the study reported here, we reduced the injected plasma volume to 50 µL. Although testing higher dilution conditions is beyond the scope of the present study, such experiments are planned to assess the influence of particle concentration on apparent dimerization. In addition, while our targeted LC-MS/MS assay was not designed to capture non-apoB proteins potentially associated with LDL, future untargeted proteomic analysis of AF4 fractions may help determine whether protein bridges contribute to this phenomenon. All considered, our current hypothesis is that the propensity of LDL for dimerization is associated with apoE binding to LDL and should be considered among the important features that affect LDL function in vivo.

### Comparison with NMR data

Comparison of our AF4-LC-MS/MS (hereafter MS) results with an NMR was insightful, as both platforms quantify HDL-P and LDL-P using deconvolution and volumetric calculations, albeit with different size and concentration calibration principles. The NMR platform uses empirically trained algorithms to also report apoA1 and apoB concentrations [34]. NMR’s clinical validation in large-scale studies confirmed its ability to detect significant disease-related differences in lipoprotein classes and subclasses [5, 62–66].

Overall, across 666 samples with a wide range of TG and TC levels, as well as statin use and CAD diagnosis, correlations and differences in total apoA1 and apoB between MS and NMR platforms showed good agreement, despite fundamental methodological differences. For HDL-P and LDL-P, application of our mass-diameter correction reduced MS-NMR % differences, decreased the MS-NMR correlation intercepts, and brought correlation slopes closer to unity. Importantly, these corrections derived independently by matching apoA1/HDL-P and apoB/LDL-P with consensus values that were consistent with the presence of HDL disks and LDL dimers. The improved MS-NMR match suggests that consideration of particle shape plays a critical role in volumetric Lp-P calculations and harmonization between analytical platforms.

We further infer that regrouping s-LDL particle dimers to the L-LDL size range would yield a dominant s-LDL subclass distribution similar to that reported by NMR. Direct comparison, however, is limited because AF4 fractions are too diluted for NMR analysis. We propose that the NMR-derived LDL-P measurements are less affected by LDL dimerization because NMR-based size estimation relies on the difference in magnetic susceptibility, specifically, the magnetic shielding of terminal methyl and methylene protons, which scales with particle mass diameter. Therefore, the NMR-LDL-P reflects the total number of s-LDL particles, including both monomers and dimers, in proportion to apoB molecules. However, information regarding the propensity for s-LDL dimerization is not retained in the NRM measurement.

### Strengths and limitations of the study

A possible limitation of our study is that we used samples from CATHGEN participants with heterogeneous atherosclerosis stages, including ~ 1/3 with no detectable atheroma. In addition, the origin of the CAD diagnosis may have different causes, which may complicate the analysis of patient samples as a single group. However, the common alternative approach of using more homogeneous and monodisperse HDL and LDL subspecies collected from fewer individuals also has limitations. Obtaining homogeneous and monodisperse subspecies of HDL and LDL requires the coupling of multiple orthogonal techniques involving multiple matrix exchanges and dilution steps. As a result, these homogeneous and monodisperse isolates may not be representative of the original in vivo HDL and LDL particle populations.

Our approach benefits from a minimally invasive one-step AF4 separation procedure. The average HDL and LDL measures across our randomly selected study population, including both healthy and atherosclerotic individuals, may therefore be more representative of HDL and LDL subspecies in vivo. As the samples were stored in the CATHGEN biorepository for over a decade before our analysis (2016–2018), some degradation might have occurred, including LDL oxidation, which could have promoted LDL dimerization. However, the strong concordance of HDL-P, LDL-P, total apoB, and apoA1 between the NMR and AF4-LC-MS platforms suggests that the sample quality remained acceptable.

While this report emphasizes only the significant influence of total TG concentrations, our data also allow the examination of potential confounding effects from age, sex, hypertension, diabetes, smoking, and statin use, which will be reported elsewhere. We also suspect additional sources of bias due to the binding of large glycoprotein in plasma, indicative of inflammation, which are beyond the scope of this report but under investigation in our laboratory.

### Perspectives

Quantifying lipoproteins as particle-like functional entities remains an analytical challenge due to the presence of numerous HDL and LDL subclasses that must be assessed amid confounding differences in size, composition, and particle numbers. Because of this complexity, subclass data are often oversimplified by using imprecise relative terms, such as “size shifts” of individual proteins and lipids, or more or less “lipidation” of apoA1 and apoB. In this study, we describe size profiles in terms of subspecies representing a similar set of proteins and lipids but with different relative composition and particle number. Converting deconvoluted concentration profiles of lipid and protein constituents into combined molecular volumes and their ratios revealed structural features such as surface thickness, core size, and contribution of embedded protein volume to the phospholipid monolayer. In this framework, qualitative descriptors such as “size shift” and “lipidation” were translated into quantitative metrics, including the relative concentration of size-defined subspecies that form spheres, disks, and dimers.

Our findings support the continued focus on standardizing clinical methodologies for lipoprotein measurements around apoA1 and apoB [33, 67, 68]. HDL and LDL subclass definitions should be validated and harmonized based on quantitative assessment of composition and structural characteristics instead of strictly enforced size cutoffs applied across different analytical platforms.

## Supplementary Information

Below is the link to the electronic supplementary material.

Supplementary file1 (PDF 3.38 MB)

## Data Availability

The datasets used and/or analyzed during the current study are contained within the manuscript and are available from the corresponding author upon reasonable request.

## Declarations

### Source of biological material

Human plasma samples were obtained from the CATHGEN biorepository at Duke University Medical Center, collected during diagnostic coronary artery catheterization procedures, under Institutional Review Board approval and with informed consent from all participants.

## References

[1] Schneider H, Morrod RS, Colvin JR, Tattrie NH. The lipid core model of lipoproteins. Chem Phys Lipids. 1973;10(4):328–53. 10.1016/0009-3084(73)90058-3. 4354881 10.1016/0009-3084(73)90058-3

[2] Shen BW, Scanu AM, Kezdy FJ. Structure of human serum lipoproteins inferred from compositional analysis. Proc Natl Acad Sci U S A. 1977;74(3):837–41. 265578 10.1073/pnas.74.3.837 PMC430495

[3] Lund-Katz S, Phillips MC. Location and motion of free cholesterol molecules in high density lipoprotein. Biochem Biophys Res Commun. 1981;100(4):1735–42. 10.1016/0006-291X(81)90719-1. 7295323 10.1016/0006-291x(81)90719-1

[4] Segrest JP, Jones MK, De Loof H, Brouillette CG, Venkatachalapathi YV, Anantharamaiah GM. The amphipathic helix in the exchangeable apolipoproteins: a review of secondary structure and function. J Lipid Res. 1992;33(2):141–66. 1569369

[5] Warnick GR, McNamara JR, Boggess CN, Clendenen F, Williams PT, Landolt CC. Polyacrylamide gradient gel electrophoresis of lipoprotein subclasses. Clin Lab Med. 2006;26(4):803. 10.1016/j.cll.2006.07.005. 17110241 10.1016/j.cll.2006.07.005

[6] Warnick GR, Kimberly MM, Waymack PP, Leary ET, Myers GL. Standardization of measurements for cholesterol, triglycerides, and major lipoproteins. Labmedicine. 2008;39(8):481–90. 10.1309/6ul9rhjh1jffu4py.

[7] Ensign W, Hill N, Heward CB. Disparate LDL phenotypic classification among 4 different methods assesing LDL particle characteristics. Clin Chem. 2006;52(9):1722–7. 10.1373/clinchem.2005.059949. 16740651 10.1373/clinchem.2005.059949

[8] Arsenault BJ, Lemieux I, Després JP, Wareham NJ, Stroes ESG, Kastelein JJP, Khaw KT, Boekholdt SM. Comparison between gradient gel electrophoresis and nuclear magnetic resonance spectroscopy in estimating coronary heart disease risk associated with LDL and HDL particle size. Clin Chem. 2010;56(5):789–98. 10.1373/clinchem.2009.140939. 20348400 10.1373/clinchem.2009.140939

[9] Hopkins PN, Pottala JV, Nanjee MN. A comparative study of four independent methods to measure LDL particle concentration. Atherosclerosis. 2015;243(1):99–106. 10.1016/j.atherosclerosis.2015.08.042. 26363807 10.1016/j.atherosclerosis.2015.08.042

[10] Miller WG, Myers GL, Sakurabayashi I, Bachmann LM, Caudill SP, Dziekonski A, Edwards S, Kimberly MM, Korzun WJ, Leary ET, Nakajima K, Nakamura M, Nilsson G, Shamburek RD, Vetrovec GW, Warnick GR, Remaley AT. Seven direct methods for measuring HDL and LDL cholesterol compared with ultracentrifugation reference measurement procedures. Clin Chem. 2010;56(6):977–86. 10.1373/clinchem.2009.142810. 20378768 10.1373/clinchem.2009.142810 PMC4687457

[11] Rosenson RS, Brewer J, Bryan H, Chapman MJ, Fazio S, Hussain MM, Kontush A, Krauss RM, Otvos JD, Remaley AT, Schaefer EJ. HDL Measures, particle heterogeneity, proposed nomenclature, and relation to atherosclerotic cardiovascular events. Clin Chem. 2011;57(3):392–410. 10.1373/clinchem.2010.155333. 21266551 10.1373/clinchem.2010.155333

[12] McNamara JR, Russell Warnick G, Cooper GR. A brief history of lipid and lipoprotein measurements and their contribution to clinical chemistry. Clin Chim Acta. 2006;369(2):158–67. 10.1016/j.cca.2006.02.041. 16740255 10.1016/j.cca.2006.02.041

[13] Rafai N, Warnick RG, Dominiczak. Handbook of Lipoprotein Testing. 2nd edition edn. AACC Press, Washington, DC. 2000;21:609-623. 10.1093/clinchem/47.2.359a

[14] Mora S, Szklo M, Otvos JD, Greenland P, Psaty BM, Goff DC, O’Leary DH, Saad MF, Tsai MY, Sharrett AR. LDL particle subclasses, LDL particle size, and carotid atherosclerosis in the Multi-Ethnic Study of Atherosclerosis (MESA). Atherosclerosis. 2007;192(1):211–7. 16765964 10.1016/j.atherosclerosis.2006.05.007

[15] Stock EO, Asztalos BF, Miller JM, He L, Creasy KT, Schwemberger R, Quinn A, Pullinger CR, Malloy MJ, Diffenderfer MR, Kane JP. High-density lipoprotein particles, inflammation, and coronary heart disease risk. Nutrients. 2025. 10.3390/nu17071182. 40218941 10.3390/nu17071182 PMC11990870

[16] Lu M, Gursky O. Aggregation and fusion of low-density lipoproteins in vivo and in vitro. Biomol Concepts. 2013;4(5):501–18. 10.1515/bmc-2013-0016. 25197325 10.1515/bmc-2013-0016 PMC4154560

[17] Pentikainen MO, Lehtonen EMP, Kovanen PT. Aggregation and fusion of modified low density lipoprotein. J Lipid Res. 1996;37(12):2638–49. 9017515

[18] Ruuth M, Nguyen SD, Vihervaara T, Hilvo M, Laajala TD, Kondadi PK, Gisterå A, Lähteenmäki H, Kittilä T, Huusko J, Uusitupa M, Schwab U, Savolainen MJ, Sinisalo J, Lokki ML, Nieminen MS, Jula A, Perola M, Ylä-Herttula S, Rudel L, Öörni A, Baumann M, Baruch A, Laaksonen R, Ketelhuth DFJ, Aittokallio T, Jauhiainen M, Käkelä R, Borén J, Williams KJ, Kovanen PT, Öörni K. Susceptibility of low-density lipoprotein particles to aggregate depends on particle lipidome, is modifiable, and associates with future cardiovascular deaths. Eur Heart J. 2018;39(27):2562–73. 10.1093/eurheartj/ehy319. 29982602 10.1093/eurheartj/ehy319 PMC6047440

[19] Davidson WS. HDL-C vs HDL-P: how changing one letter could make a difference in understanding the role of high-density lipoprotein in disease. Clin Chem. 2014;60(11):e1–3. 10.1373/clinchem.2014.232769. 25281702 10.1373/clinchem.2014.232769

[20] Bermudez-Lopez M, Perpiñan H, Amigo N, Castro E, Alonso N, Mauricio D, Fernandez E, Valdivielso JM. Advanced lipoprotein parameters could better explain atheromatosis in non-diabetic chronic kidney disease patients. Clin Kidney J. 2021;14(12):2591–9. 10.1093/ckj/sfab113. 34950470 10.1093/ckj/sfab113 PMC8690051

[21] Caulfield MP, Li S, Lee G, Blanche PJ, Salameh WA, Benner WH, Reitz RE, Krauss RM. Direct determination of lipoprotein particle sizes and concentrations by ion mobility analysis. Clin Chem. 2008;54(8):1307–16. 10.1373/clinchem.2007.100586. 18515257 10.1373/clinchem.2007.100586

[22] Vaisar T, Kanter JE, Wimberger J, Irwin AD, Gauthier J, Wolfson E, Bahnam V, Wu IH, Shah H, Keenan HA, Greenbaum CJ, King GL, Heinecke JW, Bornfeldt KE. High concentration of medium-sized HDL particles and enrichment in HDL paraoxonase 1 associate with protection from vascular complications in people with long-standing type 1 diabetes. Diabetes Care. 2020;43(1):178–86. 10.2337/dc19-0772. 31597668 10.2337/dc19-0772 PMC6925582

[23] Hutchins PM, Ronsein GE, Monette JS, Pamir N, Wimberger J, He Y, Anantharamaiah GM, Kim DS, Ranchalis JE, Jarvik GP, Vaisar T, Heinecke JW. Quantification of HDL particle concentration by calibrated ion mobility analysis. Clin Chem. 2014;60(11):1393–401. 10.1373/clinchem.2014.228114. 25225166 10.1373/clinchem.2014.228114 PMC4324763

[24] Lounila J, Ala-Korpela M, Jokisaari J, Savolainen MJ, Kesäniemi YA. Effects of orientational order and particle size on the NMR line positions of lipoproteins. Phys Rev Lett. 1994;72(25):4049–52. 10.1103/PhysRevLett.72.4049. 10056366 10.1103/PhysRevLett.72.4049

[25] Otvos JD, Jeyarajah EJ, Bennett DW. Quantification of plasma-lipoproteins by proton nuclear-magnetic-resonance spectroscopy. Clin Chem. 1991;37(3):377–86. 2004444

[26] Segrest JP, Cheung MC, Jones MK. Volumetric determination of apolipoprotein stoichiometry of circulating HDL subspecies. J Lipid Res. 2013;54(10):2733–44. 10.1194/jlr.M039172. 23883582 10.1194/jlr.M039172 PMC3770086

[27] McNamara JR, Small DM, Li ZL, Schaefer EJ. Differences in LDL subspecies involve alterations in lipid composition and conformational changes in apolipoprotein B. J Lipid Res. 1996;37(9):1924–35. 8895058

[28] Kuklenyik Z, Jones JI, Gardner MS, Schieltz DM, Parks BA, Toth CA, Rees JC, Andrews ML, Carter K, Lehtikoski AK, McWilliams LG, Williamson YM, Bierbaum KP, Pirkle JL, Barr JR. Core lipid, surface lipid and apolipoprotein composition analysis of lipoprotein particles as a function of particle size in one workflow integrating asymmetric flow field-flow fractionation and liquid chromatography-tandem mass spectrometry. PLoS ONE. 2018. 10.1371/journal.pone.0194797. 29634782 10.1371/journal.pone.0194797 PMC5892890

[29] Kuklenyik Z, Gardner M, Parks BA, Schieltz DM, Rees JC, McWilliams LG, Williamson YM, Pirkle JL, Barr JR. Multivariate DoE optimization of asymmetric flow field flow fractionation coupled to quantitative LC-MS/MS for analysis of lipoprotein subclasses. Chromatography. 2015;2:96–117.

[30] Kraus WE, Granger CB, Sketch MH Jr., Donahue MP, Ginsburg GS, Hauser ER, Haynes C, Newby LK, Hurdle M, Dowdy ZE, Shah SH. A guide for a cardiovascular genomics biorepository: the CATHGEN experience. J Cardiovasc Transl Res. 2015;8(8):449–57. 10.1007/s12265-015-9648-y. 26271459 10.1007/s12265-015-9648-y PMC4651812

[31] Jeyarajah EJ, Cromwell WC, Otvos JD. Lipoprotein particle analysis by nuclear magnetic resonance spectroscopy. Clin Lab Med. 2006;26(4):847–70. 10.1016/j.cll.2006.07.006. 17110242 10.1016/j.cll.2006.07.006

[32] Balling M, Langsted A, Afzal S, Varbo A, Davey Smith G, Nordestgaard BG. A third of nonfasting plasma cholesterol is in remnant lipoproteins: lipoprotein subclass profiling in 9293 individuals. Atherosclerosis. 2019;286:97–104. 10.1016/j.atherosclerosis.2019.05.011. 31108411 10.1016/j.atherosclerosis.2019.05.011

[33] Delatour V, Clouet-Foraison N, Gaie-Levrel F, Marcovina SM, Hoofnagle AN, Kuklenyik Z, Caulfield MP, Otvos JD, Krauss RM, Kulkarni KR, Contois JH, Remaley AT, Vesper HW, Cobbaert CM, Gillery P. Comparability of lipoprotein particle number concentrations across ES-DMA, NMR, LC-MS/MS, immunonephelometry, and VAP: in search of a candidate reference measurement procedure for apoB and non-HDL-P standardization. Clin Chem. 2018;64(10):1485–95. 10.1373/clinchem.2018.288746. 30087138 10.1373/clinchem.2018.288746

[34] Garcia E, Bennett DW, Connelly MA, Jeyarajah EJ, Warf FC, Shalaurova I, Matyus SP, Wolak-Dinsmore J, Oskardmay DN, Young RM, Sampson M, Remaley AT, Otvos JD. The extended lipid panel assay: a clinically-deployed high-throughput nuclear magnetic resonance method for the simultaneous measurement of lipids and Apolipoprotein B. Lipids Health Dis. 2020;19 (1). 10.1186/s12944-020-01424-2 10.1186/s12944-020-01424-2 PMC7709389 33261644

[35] Kim KH, Lee JY, Lim S, Moon MH. Top-down lipidomic analysis of human lipoproteins by chip-type asymmetrical flow field-flow fractionation-electrospray ionization-tandem mass spectrometry. J Chromatogr A. 2013;1280:92–7. 10.1016/j.chroma.2013.01.025. 23375771 10.1016/j.chroma.2013.01.025

[36] Rambaldi DC, Reschiglian P, Zattoni A, Johann C. Enzymatic determination of cholesterol and triglycerides in serum lipoprotein profiles by asymmetrical flow field-flow fractionation with on-line, dual detection. Anal Chim Acta. 2009;654(1):64–70. 10.1016/j.aca.2009.06.016. 19850170 10.1016/j.aca.2009.06.016

[37] Qureshi RN, Kok WT, Schoenmakers PJ. Fractionation of human serum lipoproteins and simultaneous enzymatic determination of cholesterol and triglycerides. Anal Chim Acta. 2009;654(1):85–91. 19850173 10.1016/j.aca.2009.06.060

[38] Toth CA, Kuklenyik Z, Jones JI, Parks BA, Gardner MS, Schieltz DM, Rees JC, Andrews ML, McWilliams LG, Pirkle JL, Barr JR. On-column trypsin digestion coupled with LC-MS/MS for quantification of apolipoproteins. J Proteomics. 2017;150:258–67. 10.1016/j.jprot.2016.09.011. 27667389 10.1016/j.jprot.2016.09.011 PMC10071838

[39] Kuklenyik Z, Jones JI, Toth CA, Gardner MS, Pirkle JL, Barr JR. Optimization of the linear quantification range of an online trypsin digestion coupled liquid chromatography–tandem mass spectrometry (LC–MS/MS) platform. Instrum Sci Technol. 2017;46(1):102. 10.1080/10739149.2017.1311912. 37180980 10.1080/10739149.2017.1311912 PMC10174070

[40] Gardner MS, McWilliams LG, Jones JI, Kuklenyik Z, Pirkle JL, Barr JR. Simultaneous quantification of free cholesterol, cholesteryl esters, and triglycerides without ester hydrolysis by UHPLC separation and in-source collision induced dissociation coupled MS/MS. J Am Soc Mass Spectrom. 2017. 10.1007/s13361-017-1756-2. 28801822 10.1007/s13361-017-1756-2 PMC5645443

[41] Gardner MS, Kuklenyik Z, Lehtikoski A, Carter KA, McWilliams LG, Kusovschi J, Bierbaum K, Jones JI, Rees J, Reis G, Pirkle JL, Barr JR. Development and application of a high throughput one-pot extraction protocol for quantitative LC-MS/MS analysis of phospholipids in serum and lipoprotein fractions in normolipidemic and dyslipidemic subjects. J Chromatogr, B: Anal Technol Biomed Life Sci. 2019;1118:137–47. 10.1016/j.jchromb.2019.04.041. 10.1016/j.jchromb.2019.04.041 PMC7138451 31035135

[42] Zhang L, Song J, Cavigiolio G, Ishida BY, Zhang S, Kane JP, Weisgraber KH, Oda MN, Rye KA, Pownall HJ, Ren G. Morphology and structure of lipoproteins revealed by an optimized negative-staining protocol of electron microscopy. J Lipid Res. 2011;52(1):175–84. 10.1194/jlr.D010959. 20978167 10.1194/jlr.D010959 PMC2999936

[43] Erickson HP. Size and shape of protein molecules at the nanometer level determined by sedimentation, gel filtration, and electron microscopy. Biol Proced Online. 2009;11(1):32–51. 19495910 10.1007/s12575-009-9008-x PMC3055910

[44] Sakurai T, Trirongjitmoah S, Nishibata Y, Namita T, Tsuji M, Hui SP, Jin S, Shimizu K, Chiba H. Measurement of lipoprotein particle sizes using dynamic light scattering. Ann Clin Biochem. 2010;47:476–81. 20736248 10.1258/acb.2010.010100

[45] Johnson CS, Gabriel DA (2018) Laser light scattering. In: Spectroscopy in Biochemistry, vol 2. CRC Press, pp 177–272. 10.1201/9781351076814

[46] Dahneke BE, Hutchins DK. Characterization of particles by modulated dynamic light scattering. I. Theory. J Chem Phys. 1994;100(11):7890–902. 10.1063/1.466835.

[47] Segrest JP. Why high density lipoprotein: phospholipid recombinants cannot be spherical micelles. FEBS Lett. 1979;106(1):169–70. 10.1016/0014-5793(79)80720-6. 227724 10.1016/0014-5793(79)80720-6

[48] Niisuke K, Kuklenyik Z, Horvath KV, Gardner MS, Toth CA, Asztalos BF. Composition-function analysis of HDL subpopulations: influence of lipid composition on particle functionality. J Lipid Res. 2020;61(3):306–15. 10.1194/jlr.RA119000258. 31953305 10.1194/jlr.RA119000258 PMC7053829

[49] Milne R, Theilis JR, Maurice R, Pease RJ, Weech PK, Rassart E, Fruchart JC, Scott J, Marcel YL. The use of monoclonal antibodies to localize the low density lipoprotein receptor-binding domain of apolipoprotein B. J Biol Chem. 1989;264(33):19754–60. 2479639

[50] Milne RW, Marcel YL. Monoclonal antibodies against human low density lipoprotein. Stoichiometric binding studies using Fab fragments. FEBS Lett. 1982;146(1):97–100. 10.1016/0014-5793(82)80712-6. 6183147 10.1016/0014-5793(82)80712-6

[51] Liu Y, Atkinson D. Immuno-electron cryo-microscopy imaging reveals a looped topology of apoB at the surface of human LDL. J Lipid Res. 2011;52(6):1111–6. 10.1194/jlr.M013946. 21460103 10.1194/jlr.M013946 PMC3090232

[52] Reimund M, Dearborn AD, Graziano G, Lei H, Ciancone AM, Kumar A, Holewinski R, Neufeld EB, O’Reilly FJ, Remaley AT, Marcotrigiano J. Structure of apolipoprotein B100 bound to the low-density lipoprotein receptor. Nature. 2025;638(8051):829–35. 10.1038/s41586-024-08223-0. 39663455 10.1038/s41586-024-08223-0

[53] Berneis KK, Krauss RM. Metabolic origins and clinical significance of LDL heterogeneity. J Lipid Res. 2002;43(9):1363–79. 10.1194/jlr.R200004-JLR200. 12235168 10.1194/jlr.r200004-jlr200

[54] Grundy SM, Stone NJ, Bailey AL, Beam C, Birtcher KK, Blumenthal RS, Braun LT, de Ferranti S, Faiella-Tommasino J, Forman DE, Goldberg R, Heidenreich PA, Hlatky MA, Jones DW, Lloyd-Jones D, Lopez-Pajares N, Ndumele CE, Orringer CE, Peralta CA, Saseen JJ, Smith SC Jr, Sperling L, Virani SS, Yeboah J. 2018 AHA/ACC/AACVPR/AAPA/ABC/ACPM/ADA/AGS/APhA/ASPC/NLA/PCNA guideline on the management of blood cholesterol: executive summary: a report of the American College of Cardiology/American Heart Association Task Force on Clinical Practice Guidelines. J Am Coll Cardiol. 2019;73(24):3168–209. 10.1016/j.jacc.2018.11.002. 30423391 10.1016/j.jacc.2018.11.002

[55] Zou P, Ting AY. Imaging LDL receptor oligomerization during endocytosis using a co-internalization assay. ACS Chem Biol. 2011;6(4):308–13. 10.1021/cb100361k. 21194239 10.1021/cb100361k PMC3078185

[56] Teerlink T, Scheffer PG, Bakker SJL, Heine RJ. Combined data from LDL composition and size measurement are compatible with a discoid particle shape. J Lipid Res. 2004;45(5):954–66. 10.1194/jlr.M300521-JLR200. 14967822 10.1194/jlr.M300521-JLR200

[57] Maric S, Lind TK, Lyngso J, Cardenas M, Pedersen JS. Modeling small-angle X-ray scattering data for low-density lipoproteins: insights into the fatty core packing and phase transition. ACS Nano. 2017;11(1):1080–90. 10.1021/acsnano.6b08089. 28048943 10.1021/acsnano.6b08089

[58] Oliveira CL, Santos PR, Monteiro AM, Figueiredo Neto AM. Effect of oxidation on the structure of human low- and high-density lipoproteins. Biophys J. 2014;106(12):2595–605. 10.1016/j.bpj.2014.04.049. 24940777 10.1016/j.bpj.2014.04.049 PMC4070168

[59] Meyer DF, Nealis AS, Macphee CH, Groot PH, Suckling KE, Bruckdorfer KR, Perkins SJ. Time-course studies by synchrotron X-ray solution scattering of the structure of human low-density lipoprotein during Cu(2+)-induced oxidation in relation to changes in lipid composition. Biochem J. 1996;319(Pt 1):217–27. 10.1042/bj3190217. 8870672 10.1042/bj3190217 PMC1217758

[60] Ruuth M, Lahelma M, Luukkonen PK, Lorey MB, Qadri S, Sädevirta S, Hyötyläinen T, Kovanen PT, Hodson L, Yki-Järvinen H, Öörni K. Overfeeding saturated fat increases LDL (low-density lipoprotein) aggregation susceptibility while overfeeding unsaturated fat decreases proteoglycan-binding of lipoproteins. Arterioscler Thromb Vasc Biol. 2021;41(11):2823–36. 10.1161/ATVBAHA.120.315766. 34470478 10.1161/ATVBAHA.120.315766 PMC8545249

[61] Ruuth M, Janssen LGM, Äikäs L, Tigistu-Sahle F, Nahon KJ, Ritvos O, Ruhanen H, Käkelä R, Boon MR, Öörni K, Rensen PCN. LDL aggregation susceptibility is higher in healthy South Asian compared with white Caucasian men. J Clin Lipidol. 2019;13(6):910-919.e912. 10.1016/j.jacl.2019.09.011. 31753722 10.1016/j.jacl.2019.09.011

[62] Ikewaki K, Noma K, Tohyama J, Kido T, Mochizuki S. Effects of bezafibrate on lipoprotein subclasses and inflammatory markers in patients with hypertriglyceridemia - a nuclear magnetic resonance study. Int J Cardiol. 2005;101(3):441–7. 15907413 10.1016/j.ijcard.2004.03.071

[63] Otvos JD, Shalaurova I, Freedman DS, Rosenson RS. Effects of pravastatin treatment on lipoprotein subclass profiles and particle size in the PLAC-I trial. Atherosclerosis. 2002;160(1):41–8. 11755921 10.1016/s0021-9150(01)00544-5

[64] Sarzynski MA, Burton J, Rankinen T, Blair SN, Church TS, Després JP, Hagberg JM, Landers-Ramos R, Leon AS, Mikus CR, Rao DC, Seip RL, Skinner JS, Slentz CA, Thompson PD, Wilund KR, Kraus WE, Bouchard C. The effects of exercise on the lipoprotein subclass profile: a meta-analysis of 10 interventions. Atherosclerosis. 2015;243(2):364–72. 10.1016/j.atherosclerosis.2015.10.018. 26520888 10.1016/j.atherosclerosis.2015.10.018 PMC4663138

[65] Otvos JD, Mora S, Shalaurova I, Greenland P, Mackey RH, Goff JDC. Clinical implications of discordance between LDL cholesterol and LDL particle number. J Clin Lipidol. 2011;5(2):105–13. 10.1016/j.jacl.2011.02.001. 21392724 10.1016/j.jacl.2011.02.001 PMC3070150

[66] Soininen P, Kangas AJ, Würtz P, Suna T, Ala-Korpela M. Quantitative serum nuclear magnetic resonance metabolomics in cardiovascular epidemiology and genetics. Circ Cardiovasc Genet. 2015;8(1):192–206. 10.1161/CIRCGENETICS.114.000216. 25691689 10.1161/CIRCGENETICS.114.000216

[67] Ruhaak LR, Romijn FPHTM, Begcevic Brkovic I, Kuklenyik Z, Dittrich J, Ceglarek U, Hoofnagle AN, Althaus H, Angles-Cano E, Coassin S, Delatour V, Deprez L, Dikaios I, Kostner GM, Kronenberg F, Lyle A, Prinzing U, Vesper HW, Cobbaert CM. Development of an LC-MRM-MS-based candidate reference measurement procedure for standardization of serum apolipoprotein (a) tests. Clin Chem. 2023;69(3):251–61. 10.1093/clinchem/hvac204. 36644914 10.1093/clinchem/hvac204

[68] Cobbaert CM, Althaus H, Begcevic Brkovic I, Ceglarek U, Coassin S, Delatour V, Deprez L, Dikaios I, Dittrich J, Hoofnagle AN, Kostner GM, Kronenberg F, Kuklenyik Z, Prinzing U, Vesper HW, Zegers I, Ruhaak LR. Towards an SI-traceable reference measurement system for seven serum apolipoproteins using bottom-up quantitative proteomics: conceptual approach enabled by cross-disciplinary/cross-sector collaboration. Clin Chem. 2021;67(3):478–89. 10.1093/clinchem/hvaa239. 33331636 10.1093/clinchem/hvaa239

