# Supplementary material for: Effect of HDL disk and LDL dimer presence on lipoprotein particle number determination and subclassification
Source: Anal Bioanal Chem. 2026 Feb 27;418(9):2619–39. doi: 10.1007/s00216-026-06390-9 (PMC13079496; doi:10.1007/s00216-026-06390-9)
Supplement: Supplementary file 1 — Supplementary file1 (PDF 3.38 MB) [file 216_2026_6390_MOESM1_ESM.pdf]

Supplementary Information (SI)

Effect of HDL disk and LDL dimer presence on lipoprotein particle number determination and subclassification

Zsuzsanna Kuklenyik[1], Anna A. Ivanova[1], Lauren E. Drinkard[1], David M. Schieltz[1], Jeffrey I. Jones[1], Kevin Bierbaum[1], Christopher A. Toth[1], Michael S. Gardner[1], Bryan A. Parks[1], Michael Andrews[1], Jennifer D. Kusovschi[1], Jack A. Sultan[1], Antony Lehtikoski[1], Jon C. Rees[1], Wanda I. Santana[1], Victoria Connor[1], Eric C. Leszczynski[3], Robert W. McGarrah III[2], Mark A. Sarzynski[3], William E. Kraus[2], and John R. Barr[1]*

[1] Clinical Chemistry Branch, Division of Laboratory Sciences, Centers for Disease Control and Prevention, Atlanta, Georgia, United States of America

[2] Duke Molecular Physiology Institute, Duke University School of Medicine, Duke University, Durham, NC, 27701 USA

[3]University of South Carolina, 921 Assembly Street, Columbia, SC, 29201, USA

*** Corresponding author**: John R. Barr, email: jbb0@cdc.gov, mailing address: 4770 Buford Highway, Atlanta GA, 30341, phone: 1-770-488-7848; fax: 1-770-488-0509

Protein analysis method

According to a published on-line trypsin digestion method [1], A 50 µL aliquot of each fraction was diluted 3fold with buffer containing 10 mM sodium bicarbonate, 150 mM NaCl (pH 7.4), and 0.15% Zwittergent 3-12 (EMD Millipore, Billerica, MA, USA). The protein concentrations in the fractions were calculated based on external calibration using a dilution series of a serum pool, which was previously value assigned by standard addition methodology using purified protein standards. The analysis was performed with an online column switching system, Perfinity (Shimadzu Scientific Instruments/Perfinity Biosciences Inc.), equipped with a trypsin column (2.1 x 50 mm), two alternating trapping columns, and an analytical column (HALO C18 core-shell 5 x 2.1 mm 2.7 µm and 100 x 2.1 mm 2.7 µm particle size, respectively). The autosampler was programmed to pick up 50 µL from each diluted fraction and 5µL isotopically labeled peptide mix (~10 nmol/mL from a separate vial). Merged in the autosampler loop, the sample and the labeled peptides were passed through the online trypsin column with a 25 µL/min flow rate. The peptide cleavage products and labeled peptide analogs were collected together on the trapping column for 8 min, then transferred/separated on the analytical column using a 3-95% [0.1% formic acid in acetonitrile]/[0.1% formic acid in water] solvent gradient program with 350 µL/min flow rate (total run time with column equilibration was 12 min). The native peptide and the isotopically labeled peptide internal standard chromatograms were acquired in multiple reaction monitoring mode on a 6500 Qtrap instrument (AB Sciex, Foster City, CA, USA). The protein concentrations in the fractions were calculated on the basis of external calibration using a dilution series of a serum pool, which was previously value assigned by standard addition methodology using purified protein standards.

Non-polar lipid analysis method

The analysis, based on a published one-pot protocol [2], required 50 µL of a purified AF4 fraction aliquot. After protein precipitation with 200 µL 70:15:15 ethanol : methyl-t-butyl ether : dichloromethane containing internal standards, the samples were evaporated under a stream of nitrogen, with 60 °C heating plate temperature for approximately 30 min until dryness was observed by visual inspection. The dried samples were extracted/reconstituted in 50 µL of nonane. The UHPLC system was an Agilent 1290 (Agilent Technologies, Santa Clara, CA, USA). From each sample, 6 µL was injected. The column was a Kinetex HILIC 1.7 µm, 2.1 × 50 mm (Phenomenex, Torrance, CA, USA). Mobile phase A was hexanes with 0.05% isopropanol. Mobile phase B was hexanes with 5% ethanol and 0.05% isopropanol. The mobile phase flow rate was 600 µL/min. The separation was a gradient elution, starting at 2% B, increasing to 4% B over 0.5 min, then to 80% B over 1.0 min, returning to 2% B over 0.1 min, and finally holding at 2% B for 1.4 min to re-equilibrate the column. The total run time was 3 min. The mass spectrometer was a Sciex 4000 QTrap (Sciex, Framingham, MA, USA). The heated nebulizer (APCI) interface, normally operated with air as the nebulizer gas, was used with nitrogen because of the flammable nature of the volatile organic LC eluent. In-source collision-induced dissociation (CID) was employed for CE and TAG, as opposed to separate MRM transitions for each species.

Phospholipid analysis method

Utilizing a published one-pot protocol [3], the analysis, required 20 µL of a purified AF4 fraction aliquot. After protein precipitation with 180 µL 70:15:15 ethanol : methyl-t-butyl ether : dichloromethane containing internal standards, the samples were evaporated under a stream of nitrogen, with 60 °C heating plate temperature for approximately 30 min until dryness was observed by visual inspection. The dried samples were extracted/reconstituted in 200 µL of 55:43:2

nonane:isopropanol:water. The concentration of the main phospholipids (PL), as a total of phosphatidylinositols (PI), phosphatidylcholines (PC), phosphatidylethanolamines (PE), sphingomyelins (SM), and lysophosphatidylcholines (LPC) were determined by HILIC chromatography separation and PL species targeted MS/MS detection. From the supernatant in each well, 5 µL was injected into the Acquity UHPLC system (Waters, USA) equipped with a Kinetex HILIC 100 Å pore, 2.1×100mm, 1.7 µm particle column. The separation was a gradient elution with a flow rate of 0.7 mL/min. Mobile phase A was 99:1 acetonitrile : isopropanol. Mobile phase B was 2.5mM aqueous ammonium acetate in1:1 acetonitrile : water. The gradient was from 20% B to 50% B over 1.0 min, held for 0.3 min, 50% to 100% B over 0.1 min, held for 0.8 min, returned to 80% A and 20% B over 0.01 min, and held for 0.79 min. The total run time was 3.0 min. A 6500 Qtrap (Sciex, Framingham, MA) was operated in MRM scanning mode with the TurboSpray IonDrive source (ESI) installed. PI species were monitored in negative ion mode, and the remaining classes, PC, PE, SM and LPC were monitored in positive ion mode. Only typical fatty acid analogs were monitored that are present at the highest concentration in human plasma, 15 for PI, 19 for PC, 19 for PE, and 8 for LPC, using species-specific total molecular ions as precursors and generic head group product ions.

Deconvolution of size profiles

Using JMP software functions (SAS Institute Inc., USA), the experimental concentration-versus-size profiles were deconvoluted to optimize **Eq. S1**,

$$f(Q_i, \Delta, w_{1/2}, s) = \sum_{i=1}^{n} \boldsymbol{Q_i} * e^{\frac{-(d_{DLS}-s+i*\Delta)^2}{2*w_{1/2}{}^2}} \qquad \text{(S1)}$$

where the Q_i constants determined the optimal intensity of each Gaussian peak, d_{DLS} is the measured hydrodynamic size in the fractions (nm), i is the number of increments from the starting time-point s (nm), Δ is the size increment between adjacent Gaussian peaks, and $w_{½}$ is the half-peak width.

The deconvolutions were performed in JMP using the Analyze/Specialized Modeling/Nonlinear function.

The deconvolution formula in JMP code:

```
Abs( theta1 ) * Exp( -(:"Size (nm)"n - (:startG + :IncG)) ^ 2 / (2 * :width ^ 2) ) +
+ Abs( theta2 ) * Exp( -(:"Size (nm)"n - (:startG + 2 * :IncG)) ^ 2 / (2 * :width ^ 2) ) +…. +
+ Abs( theta16 ) * Exp( -(:"Size (nm)"n - (:startG + 16 * :IncG)) ^ 2 / (2 * :width ^ 2) )
```

Note that using Abs(theta) was necessary because the program sampled both positive and negative values.

The optimization, typically converged in 500-700 iteration cycles.

To assess deconvolution accuracy, the DLS-measured particle sizes were substituted into **Eq.S1**, thus the summed Gaussian profiles were compared with the raw LC-MS/MS measured (non-deconvoluted) concentration *versus* particle-size profiles by each sample. Individual Gaussian peak profiles were generated using corresponding optimal Q_i terms. To display average profiles by sample groups in **Figures S2-S8**, the DLS measured sizes were

binned (1-2 fractions per bin), and the fraction concentrations were summed by size bin before calculation of the average profile.

Agreements between the back-calculated and corresponding raw measured concentrations were tested in terms of average Deconvoluted/Non-deconvoluted accuracy (%), for the HDL and LDL size ranges and by HDL and LDL size bins (**Table S3-S6**). Deconvolution of continuous elution profiles can, in general, be sensitive to model specification, and unconstrained fitting approaches may converge to local minima or physically ambiguous solutions when multiple parameters are allowed to vary freely. To mitigate this risk, the Gaussian centers and widths were fixed. The Gaussian peak width was fixed, based on the size resolution of the AF4 separation in HDL and LDL size range. Under these conditions, the estimation of theta values was reduced to a convex non-negative least squares (NNLS) problem with a unique global solution, rather than a multi-parameter nonlinear optimization prone to local minima. Beyond standard deviations (shown in supplementary Tables S4-S7), model adequacy was evaluated using multiple complementary criteria, including inspection of overlays of the Gaussian peaks with the experimental profiles, along with the sum of the Gaussian profiles.

The concentrations of individual Gaussian subspecies were derived from the optimized Q_i values using **Eq. S2.**

$$[Deconvoluted\ Gaussian\ subspecies\ concentration] = Q_i * (\pi)^{1/2} \qquad \text{[mole/L]} \qquad (S2)$$

These deconvoluted Gaussian subspecies concentrations were used for the calculation of molecular volumes (**Figure S8**).

For comparison of raw and deconvoluted profiles of concentration ratios and molecular volume ratios (**Figures S12 and S13** and main text **Figure 2 and 3**), the Gaussian subspecies ratios were weighed with the corresponding Gaussian subspecies concentrations using **Eq. S3**:

$$[back\text{-}calculated\_ratio\_profile] = \frac{\sum_{i=1}^{n} [Gaussian\ sub\_species\_ratio]_i * Q_i * e^{\frac{-(d-s+i*\Delta)^2}{2*w_{1/2}^2}}}{\sum_{i=1}^{n} Q_i * e^{\frac{-(d-s+i*\Delta)^2}{2*w_{1/2}^2}}} \qquad (S3)$$

Calculation of molecular volumes

Total particle volumes per liter in serum were calculated using **Eq. S4-S8**, by multiplying the analyte concentrations for each Gaussian subspecies (**Eq.S2**) by the corresponding literature-reported molecular volumes: V_{FC}=0.610 nm^3, V_{CE}=1.179 nm^3, V_{TG}=1.575 nm^3, and V_{PL}=1.307 nm^3. For each protein, $V_{protein}$ was calculated by multiplying the molecular weight ($MW_{protein}$) by the partial specific volume, $\nu_{protein}$=1.212 nm^3/kDa [4,5]

$$\frac{\text{Core-lipid Volume}}{\text{L of plasma}} = (V_{CE} * [CE] * N_A) + (V_{TG} * [TG] * N_A) + (V_{FC} * N_A * [FC_{Core}]) \qquad [N_A*nm^3/L] \qquad (S4)$$

$$\frac{\text{Surface-lipid Volume}}{\text{L of plasma}} = (V_{PL} * [PL] * N_A) + (V_{FC} * [FC_{surface}] * N_A) \qquad [N_A*nm^3/L] \qquad (S5)$$

$$\frac{\text{Protein Volume}}{\text{L of plasma}} = (\sum(\text{MW}_{\text{Protein}} * v_{\text{Protein}} * [\text{Protein}] * N_A)) \quad [N_A*nm^3/L] \quad (S6)$$

[FC_{core}] and [$FC_{surface}$] were calculated assuming a partitioning coefficient of 5 (5/6 *vs.* 1/6) between polar and non-polar lipid phases [4,5].

$$[\text{FC}_{\text{core}}] = [\text{FC}] * \frac{1}{6}\frac{[\text{CE}]+[\text{TG}]}{[PL]} \quad [\text{mole/L}] \quad (S7)$$

$$[\text{FC}_{\text{surface}}] = [\text{FC}] * \left(1 - \frac{1}{6}\frac{[\text{CE}]+[\text{TG}]}{[PL]}\right) \quad [\text{mole/L}] \quad (S8)$$

Bias in the partial specific volume of protein molecules may arise from differences between globular and elongated tertiary protein structure, corresponding to a range of 1.21 to 1.27 nm^3/kDa [6]. The average molecular volumes of lipid molecules were taken as reported in the literature, established by physicochemical measurements and confirmed by molecular modeling studies [7]. Variations in chain length and number of double bonds may introduce bias in the partial specific volume of lipid species. However, in plasma, the average molecular volume of lipid species within each lipid class is dominated by a limited number of prevalent species. Consequently, the systatic bias due to inaccuracies in lipid partial specific volume is minor compared to the measurement accuracy of lipid-class concentrations, which ranges between ±5-10%.

Using **Eq. 6-9 and S6-S7**, the mass (V_m), core (V_{m_ore}), and surface volumes ($V_{m\_surface}$) were calculated using **Eq.S9-S11**

$$V_m = N_A * \frac{4\pi}{3} * \left(\frac{k*\text{d}_{\text{DLS}}-2*\text{n}*\text{w}_\text{h}}{2}\right)^3 \quad [N_A*nm^3/mole] \quad (S9)$$

$$V_{m\_core} = N_A * \frac{4\pi}{3} * \left(\frac{\text{k}*\text{d}_{\text{DLS}}-2*\text{n}*\text{w}_\text{h}-2*w_{SL}}{2}\right)^3 \quad [N_A*nm^3/mole] \quad (S10)$$

$$V_{m\_surface} = V_m - V_{m\_core} \quad [N_A*nm^3/mole] \quad (S11)$$

Calculation of core/surface and core-lipid/surface-lipid volume ratios

Determination of V_{core} required knowledge of w_{SL}, which was estimated by matching the experimental core/surface molecule volume ratios to the theoretical model-derived core/surface ratios (Figures S12 and S13).

$$\frac{\frac{\text{Core-lipid Volume}}{\text{L of plasma}}}{\frac{\text{Protein Volume}}{\text{L of plasma}} + \frac{\text{Surface-lipid Volume}}{\text{L of plasma}}} = \frac{V_{core}}{V_{surface}} \quad (S12)$$

Similarly, the proportion of lipidated protein volume in the monolayer ($\%protein_{ML}$) can be estimated by matching the experimental core-lipid/surface-lipid volume ratios to the theoretical core/surface ratios:

$$\frac{\frac{\text{Core-lipid Volume}}{\text{L of plasma}}}{\frac{\text{Surface-lipid Volume}}{\text{L of plasma}}} = \frac{V_{core}}{V_{surface}*(100-\%protein_{ML})/100} \quad (S13)$$

Lp-P calculation

The Lp-P in each fraction were calculated including all main proteins and lipid classes:

$$Lp\_P_m = \frac{\left(\frac{\text{Core-lipid Volume}}{\text{L of plasma}} + \frac{\text{Surface-lipid Volume}}{\text{L of plasma}} + \frac{\text{Protein Volume}}{\text{L of plasma}}\right)}{V_m} \quad \text{[mole/L]} \qquad \text{(S14)}$$

With the estimates of w_{SL} and V_{corr}, the Lp-P can be estimated using V_{core} from **Eq. S10** and only the core-lipid molecular volume in each fraction:

$$Lp\_P_{core} = \frac{\left(\frac{\text{Core-lipid Volume}}{\text{L of plasma}}\right)}{V_{core}} \quad \text{[mole/L]} \qquad \text{(S15)}$$

Number of molecules per particle calculations

The number of analyte molecules per particle values were derived using the individual analyte concentrations divided by Lp-P values from **Eq. S14:**

$$\text{Molecules per particle} = \frac{[\mathit{Fraction}\ \text{Concentration}]}{\text{Lp\_P}} \quad \text{[mole/mole]} \qquad \text{(S16)}$$

Substitutions and rearrangements in **Eq. S15** result in the following equation:

$$\text{Molecules per particle} = \frac{[\mathit{Fraction}\ \text{Concentration}] * N_A}{\left(\frac{\text{Core-lipid Volume}}{\text{L of plasma}} + \frac{\text{Surface-lipid Volume}}{\text{L of plasma}} + \frac{\text{Protein Volume}}{\text{L of plasma}}\right)} * \left(\frac{d_m}{2}\right)^3 * \frac{4}{3} * \pi \qquad \text{(S17)}$$

Eq. S17 shows that N_A cancels out, and the effects of the AF4-Recoveries are minimal because it similarly affects both the numerator and denominator. Furthermore, **Eq. S17** highlights that the molecule per particle values is strongly affected by the accuracy of d_m which is included as $(d_m)^3$. Since d_m is estimated based on the measured d_{DLS}, maximizing the accuracy of d_{DLS} measurements by fractionation in small volume increments was highly important, i.e. collection of 40 fractions from each plasma sample.

References

1. Toth CA, Kuklenyik Z, Jones JI, Parks BA, Gardner MS, Schieltz DM, Rees JC, Andrews ML, McWilliams LG, Pirkle JL, Barr JR (2017) On-column trypsin digestion coupled with LC-MS/MS for quantification of apolipoproteins. J Proteomics 150:258-267. doi:10.1016/j.jprot.2016.09.011
2. Gardner MS, McWilliams LG, Jones JI, Kuklenyik Z, Pirkle JL, Barr JR (2017) Simultaneous Quantification of Free Cholesterol, Cholesteryl Esters, and Triglycerides without Ester Hydrolysis by UHPLC Separation and In-Source Collision Induced Dissociation Coupled MS/MS. Journal of The American Society for Mass Spectrometry. doi:10.1007/s13361-017-1756-2
3. Gardner MS, Kuklenyik Z, Lehtikoski A, Carter KA, McWilliams LG, Kusovschi J, Bierbaum K, Jones JI, Rees J, Reis G, Pirkle JL, Barr JR (2019) Development and application of a high throughput one-pot extraction protocol for quantitative LC-MS/MS analysis of phospholipids in serum and lipoprotein fractions in normolipidemic and dyslipidemic subjects. Journal of Chromatography B: Analytical Technologies in the Biomedical and Life Sciences 1118-1119:137-147. doi:10.1016/j.jchromb.2019.04.041
4. Segrest JP, Cheung MC, Jones MK (2013) Volumetric determination of apolipoprotein stoichiometry of circulating HDL subspecies. J Lipid Res 54 (10):2733-2744. doi:10.1194/jlr.M039172

5. McNamara JR, Cohn JS, Wilson PWF, Schaefer EJ (1990) Calculated values for low-density lipoprotein cholesterol in the assessment of lipid abnormalities and coronary disease risk. Clin Chem 36 (1):36-42
6. Erickson HP (2009) Size and shape of protein molecules at the nanometer level determined by sedimentation, gel filtration, and electron microscopy. Biol Proced Online 11 (1):32-51
7. Pan L, Segrest JP (2016) Computational studies of plasma lipoprotein lipids. Biochimica et Biophysica Acta - Biomembranes. doi:10.1016/j.bbamem.2016.03.010

Supplementary Tables

Table S1. Donor characteristics. The bottom sections show the number of samples stratified by TG concentrations that are used in figures throughout.

Variable	Values
Demographics	
Number of samples	666
Age, years	63 ± 12
Body Mass Index (BMI) kg/m^2	30 ± 7
Sex: male/female	407 (61%) / 259 (39%)
Race: white/black/other	480 (72%) / 142 (21%) / 44 (7%)
Conditions	
Cardiovascular disease (CAD): no/yes	225 (34%) / 441 (66%)
Diabetes mellitus (DM): no/yes	461 (69%) / 205 (31%)
Hypertension (BP): no/yes	220 (33%) / 446 (67%)
Current Smokers (SMK): no/yes	339 (51%) / 327 (49%)
Total TG concentration ranges:	(n)
<50 mg/dL	92
50-100 mg/dL	251
100-150 mg/dL	154
150-300 mg/dL	146
>300 mg/dL	23

Values are number (percentage) or mean ±SD

Table S2. Mean size measurement by dynamic light scattering at profile maxima of proteins from 256 independent runs of a QC pool. The columns indicate the Mean (Average), CV (Coefficient of Variation), and the Std Dev (Standard Deviation). A1AT, VTDB and TF were used as internal size reference in each unknown.

**Protein Name**	**Mean Size (nm)**	**%CV**	**Std Dev (nm)**
A1AT	7.5	4.5	0.3
VTDB	7.9	3.7	0.3
TF	8.4	4.8	0.4
apoA1	8.8	5.9	0.5
PLTP	12.5	8.2	1.0
HP	13.0	8.7	1.1
apoB	22.0	5.0	1.1

Table S3. Total plasma concentration of analytes (mM) by total TG concentration groups.

Analyte	TG <=50 mg/dL (n=92)	TG 50-100 mg/dL (n=251)	TG 100-150 mg/dL (n=154)	TG 150-300 mg/dL (n=146)	TG >300 mg/dL (n-23)
apoA1	44.8 (10.3)	42.1 (9.1)	40.7 (8180.5)	41 (10178.3)	40 (7.2)
apoA2	32.3 (9.9)	31.6 (8.6)	32.3 (8.7)	33 (9.1)	33.9 (5.9)
apoB	1.57 (0.53)	1.66 (0.46)	1.86 (0.49)	2.11 (0.66)	2.2 (0.89)
apoC1	8.2 (2.44)	8.05 (2,22)	8.27 (2.26)	9.54 (2.45)	13.99 (3.89)
apoC2	3.26 (1.18)	3.61 (1.29)	4.13 (1.40)	5.5 (1.72)	9.16 (3.07)
apoC3	5.12 (1.81)	5.89 (2.07)	6.76 (2.32)	8.82 (2.67)	13.94 (4.37)
apoE	1.87 (0.69)	2 (0.76)	2.02 (0.64)	2.51 (0.94)	4.17 (1.73)
FC	1108 (271)	1140 (246)	1215 (249)	1401 (322)	1779 (371)
CE	2641 (618)	2625 (608)	2787 (642)	3100 (781)	3383 (806)
TG	430 (83)	838 (163)	1380 (161)	2236 (443)	4938 (1650)
PC	2237 (484)	2312 (453)	2481 (451)	2750 (512)	3532 (590)
SM	555 (116)	549 (118)	566 (108)	586 (141)	631 (105)
PE	163 (66)	210 (86)	267 (134)	319 (133)	464 (117)
PI	43 (12)	44 (12)	50 (11)	55 (12)	75 (18)
LPC	263 (67)	255 (65)	261 (60)	272 (62)	316 (74)

Table S4. Average accuracy of HDL profile deconvolution in samples grouped based on total TG. Percent accuracy of total HDL Gaussian Peak concentrations relative to the measured concentration based on summing 20 fractions across in 6.5-15 nm size ranges (corresponding to data shown in Figure S3-S5).

Mean % (SD) in Groups by total TG						Mean % (SD) for All Samples
Analyte	<50 mg/dL (n=92)	50-100 mg/dL (n=251)	100-150 mg/dL (n=154)	150-300 mg/dL (n=146)	>300 mg/dL (n-23)	% [Sum of All Gaussian Points]/ [Sum of All Data Points] (n=666)
apoA1	95 (15)	99 (16)	101 (16)	105 (20)	107 (13)	103 (17)
apoA2	98 (17)	102 (18)	103 (17)	105 (19)	103 (11)	105 (18)
apoC1	99 (16)	102 (17)	103 (17)	105 (19)	104 (11)	101 (14)
apoC2	95 (12)	97 (14)	98 (14)	101 (17)	103 (12)	98 (13)
apoC3	92 (11)	95 (13)	95 (12)	97 (15)	99 (10)	106 (16)
apoE	82 (18)	81 (18)	77 (17)	77 (17)	78 (16)	103 (8)
PC	93 (13)	97 (15)	99 (15)	102 (17)	102 (10)	103 (15)
PE	91 (13)	94 (15)	96 (15)	99 (18)	99 (11)	101 (15)
PI	89 (14)	92 (15)	95 (15)	97 (17)	99 (10)	101 (15)
SM	89 (11)	91 (13)	92 (14)	94 (15)	93 (14)	101 (13)
FC	86 (11)	87 (13)	88 (13)	91 (15)	92 (15)	100 (13)
CE	91 (13)	93 (15)	95 (16)	97 (16)	98 (16)	104 (15)
TG	217 (168)	162 (88)	149 (80)	133 (41)	115 (26)	107 (14)

Table S5. Average accuracy of HDL profile deconvolution in size bins. Calculated from the sum of Gaussian Peak concentrations relative to the measured concentration.

Analyte	7.5 ±0.375 nm	8.25 ±0.375 nm	9 ±0.375 nm	9.75 ±0.375 nm	10.5 ±0.375 nm	11.25 ±0.375 nm	12 ±0.375 nm	12.75 ±0.375 nm	13.5 ±0.375 nm	14.25 ±0.375 nm
apoA1	115.2 (40.9)	100.8 (4.3)	99.1 (5)	101.6 (7.2)	99.8 (6.3)	100.6 (8)	99.7 (5.7)	100.5 (4.8)	99.8 (2.9)	100.2 (2)
apoA2	126.8 (71.4)	99.4 (5)	101.3 (6.7)	99.2 (7.5)	102.2 (8.6)	98.6 (8.9)	101.4 (8.9)	99.6 (5.4)	100.5 (4.2)	99.9 (2.9)
apoC1	124.7 (27.6)	97.2 (7.3)	102.9 (6.5)	97.3 (9)	103.8 (8)	96.9 (8.2)	102.7 (9)	98.6 (5.9)	101.2 (5)	99.6 (5.5)
apoC2	160.4 (224.4)	97.9 (5.3)	102.3 (6.1)	98.3 (6)	102.2 (6.9)	98 (6.8)	101.9 (6.2)	99 (5.3)	101 (5.4)	99.7 (3.3)
apoC3	201 (318.7)	97 (6.2)	102.7 (6.2)	98.1 (5.6)	102 (5.5)	98.3 (6.3)	101.5 (5.5)	99.1 (5.1)	100.9 (4.5)	99.6 (3.8)
apoE	107.5 (16.7)	100.2 (9.2)	101.2 (7.5)	100.5 (5.2)	100.3 (4.4)	100.5 (5)	99.9 (3.1)	100.4 (6.9)	100 (2.4)	100 (2.2)
LPC	107.4 (13.5)	96.3 (8.1)	103.1 (6.2)	98.4 (5.7)	101.5 (5)	99.2 (2.8)	100.3 (1.5)	99.9 (1.2)	100.1 (0.5)	100 (0.2)
PC	110.9 (20)	99.7 (4.1)	100.6 (5.2)	99.7 (5.6)	100.8 (5.4)	99.3 (6.3)	100.6 (5)	100 (4.3)	100.1 (2.8)	100.1 (2.1)
PE	115.2 (26.8)	99.5 (4.7)	101 (6.3)	99.5 (6.7)	101.4 (7.2)	99 (6.9)	100.7 (5.6)	99.9 (4.5)	100.9 (10.8)	100.3 (5.5)
PI	113.7 (34.1)	99.6 (4.6)	100.6 (5.6)	100 (6.9)	101 (7.1)	99.6 (8.1)	102.1 (18.8)	101.4 (14.9)	102.1 (17.8)	103.7 (29.4)
SM	110.4 (63.9)	99.8 (4)	100.3 (5)	99.9 (5.6)	100.6 (6.7)	99.4 (5.7)	100.9 (7.7)	100.3 (7.3)	100.1 (3.4)	100.6 (9.5)
CE	117 (28.9)	99.3 (4.4)	100.7 (5.2)	99.3 (5.7)	101.1 (5.7)	98.9 (5.7)	101 (5.8)	99.6 (4.3)	100.3 (3.1)	100.1 (3.1)
FC	109.2 (15.3)	99.3 (3.9)	100.6 (4.3)	99.5 (4.7)	101 (5)	99.1 (4.7)	100.7 (3.7)	99.7 (2.7)	100.3 (2.1)	99.9 (1.4)
TG	92.3 (12.9)	98.4 (9.1)	98.6 (9.1)	96.4 (10.9)	95 (10.2)	95.6 (10.2)	94.2 (10.8)	97.2 (10.9)	95.5 (10.6)	94.5 (14.2)

Table S6. Average accuracy of LDL profile deconvolution in samples grouped based on total TG. Percent accuracy of total LDL Gaussian Peak concentrations relative to the measured concentration based on summing in the 18-29 nm size range (corresponding to data shown in Figure S6-S8).

	Mean % (SD) in Groups by total TG					Mean % (SD) for All Samples
Analyte	<=50 mg/dL (n=92)	50-100 mg/dL (n=251)	100-150 mg/dL (n=154)	150-300 mg/dL (n=146)	>300 mg/dL (n-23)	% [Sum of All Gaussian Points]/ [Sum of All Data Points] (n=666)
apoB	108 (21)	101 (19)	97 (24)	91 (24)	83 (30)	100 (17)
apoC1	98 (56)	101 (15)	83 (35)	76 (30)	68 (30)	93 (10)
apoC2	118 (54)	109 (21)	85 (22)	78 (21)	69 (21)	94 (15)
apoC3	154 (86)	106 (19)	92 (41)	81 (35)	69 (30)	94 (12)
apoE	68 (17)	106 (15)	70 (14)	69 (13)	69 (11)	94 (12)
PC	103 (16)	104 (18)	93 (18)	89 (20)	82 (25)	100 (15)
PE	99 (18)	101 (18)	88 (17)	84 (17)	75 (23)	96 (15)
PI	101 (19)	102 (18)	91 (17)	87 (19)	78 (24)	98 (15)
SM	107 (17)	104 (19)	95 (19)	90 (21)	83 (26)	101 (15)
CE	112 (18)	106 (19)	99 (21)	96 (30)	90 (29)	104 (16)
FC	110 (18)	104 (18)	96 (21)	92 (21)	84 (28)	101 (15)
TG	82 (20)	94 (16)	94 (23)	87 (24)	82 (32)	96 (15)

Table S7. Accuracy of LDL profile deconvolution in size ranges. Calculated from the sum of Gaussian Peak concentrations relative to the measured concentration.

Analyte	19 ±0.75 nm	20.5 ±0.75 nm	22 ±0.75 nm	23.5 ±0.75 nm	25 ±0.75 nm	26.5 ±0.75 nm	28 ±0.75 nm	29.5 ±0.75 nm
apoB	103.7 (17.7)	100.4 (7.3)	100.6 (8.4)	100.7 (16.2)	100.7 (10.3)	100.4 (8.3)	100.9 (10.9)	102.8 (28.6)
apoC1	100.8 (9.9)	101 (15.1)	100.4 (9.8)	100.6 (6.4)	101.7 (16.9)	100.4 (7.3)	101.5 (15.2)	101.3 (17.5)
apoC2	101.1 (11.1)	100.7 (9.5)	100.2 (7.4)	100.8 (6.4)	100.9 (12.1)	100.7 (8.4)	102.8 (27.9)	101.1 (14.8)
apoC3	101.3 (14.1)	100.5 (9.6)	100.2 (4.4)	101.4 (12.8)	101.2 (14.2)	101.7 (17.7)	102.3 (17)	101.7 (14.2)
apoE	101.1 (14.7)	100.8 (9.9)	100.3 (4.8)	100 (5.4)	101.1 (8.3)	99.9 (4.5)	100.9 (8.4)	100.4 (5.5)
LPC	100.4 (4.7)	100.3 (5.7)	100.2 (3)	100.2 (3.9)	100.1 (5)	100.2 (5.5)	99.9 (0.8)	100.1 (0.9)
PC	100.3 (7.5)	100.1 (2.8)	100.1 (2.6)	100.2 (2.8)	100.5 (5)	100.6 (14.3)	101.4 (12.6)	100.9 (8.3)
PE	101.1 (10.1)	100.3 (7.5)	100.4 (6.1)	100.4 (3.5)	100.6 (12.8)	100.5 (6.4)	101.6 (14.3)	101.1 (9.8)
PI	101.1 (13.8)	100.9 (16.7)	100.3 (7.4)	100.2 (4.1)	101.2 (9.9)	100.3 (9.9)	102.7 (22.4)	100.6 (7.1)
SM	100.5 (6)	100.1 (2.9)	100 (2.3)	100.3 (3.1)	101.5 (21.4)	100.8 (13.1)	101 (9.8)	101.1 (7.7)
CE	101.5 (15.1)	100.5 (6.5)	100 (2.3)	100.3 (3.1)	100.1 (3.7)	100.4 (5.6)	101.2 (10.9)	101.3 (11.2)
FC	100.8 (7.2)	100.2 (5.9)	100 (1.9)	100.3 (2.8)	100.1 (3.4)	100.4 (6.9)	101.1 (9.7)	101.4 (12.4)
TG	101.4 (8.5)	99.9 (5.3)	99.9 (2.1)	100.2 (2.8)	100.1 (3.3)	100.3 (5)	100.4 (5.5)	100.8 (7.2)

Supplementary Figures

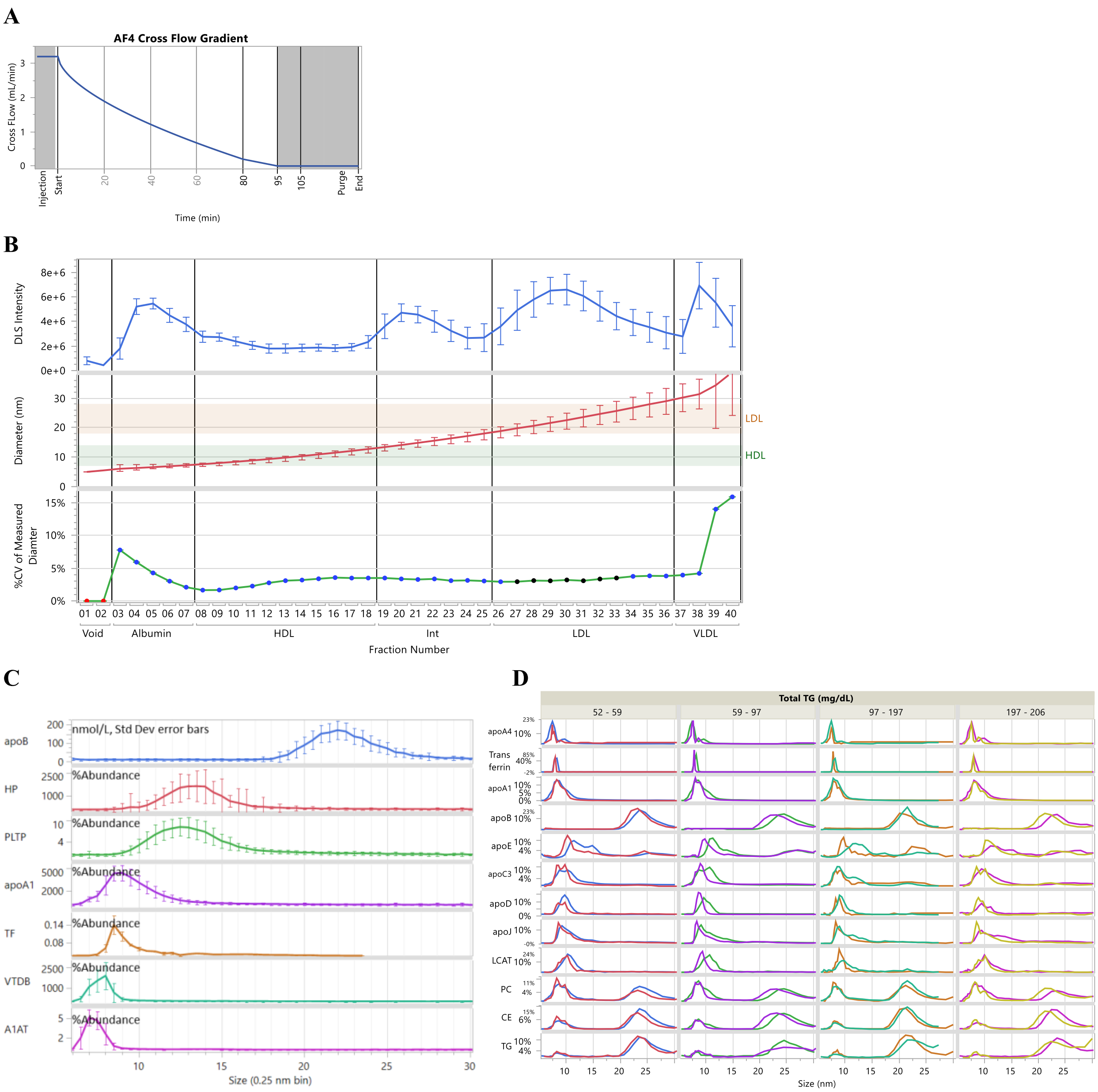

Figure S1. AF4 cross flow gradient (A). DLS signal intensity and hydrodynamic size in fraction measured by dynamic light scattering (B), and average size distribution profiles for selected proteins (C) from 256 independent runs of a QC plasma pool. Error bars indicate standard deviation. Overlay of %Abundances of eight representative samples with various total TG concentrations (D). Sample pool separated after storage at 5 °C and -80 °C for 1 day, analyzed on the same day in the same batch by AF4-LC-MS/MS workflow (E next page). One freeze-thaw cycle was a minor effect relative to other inter-day method variations (C), and differences between individual unknown samples (D).

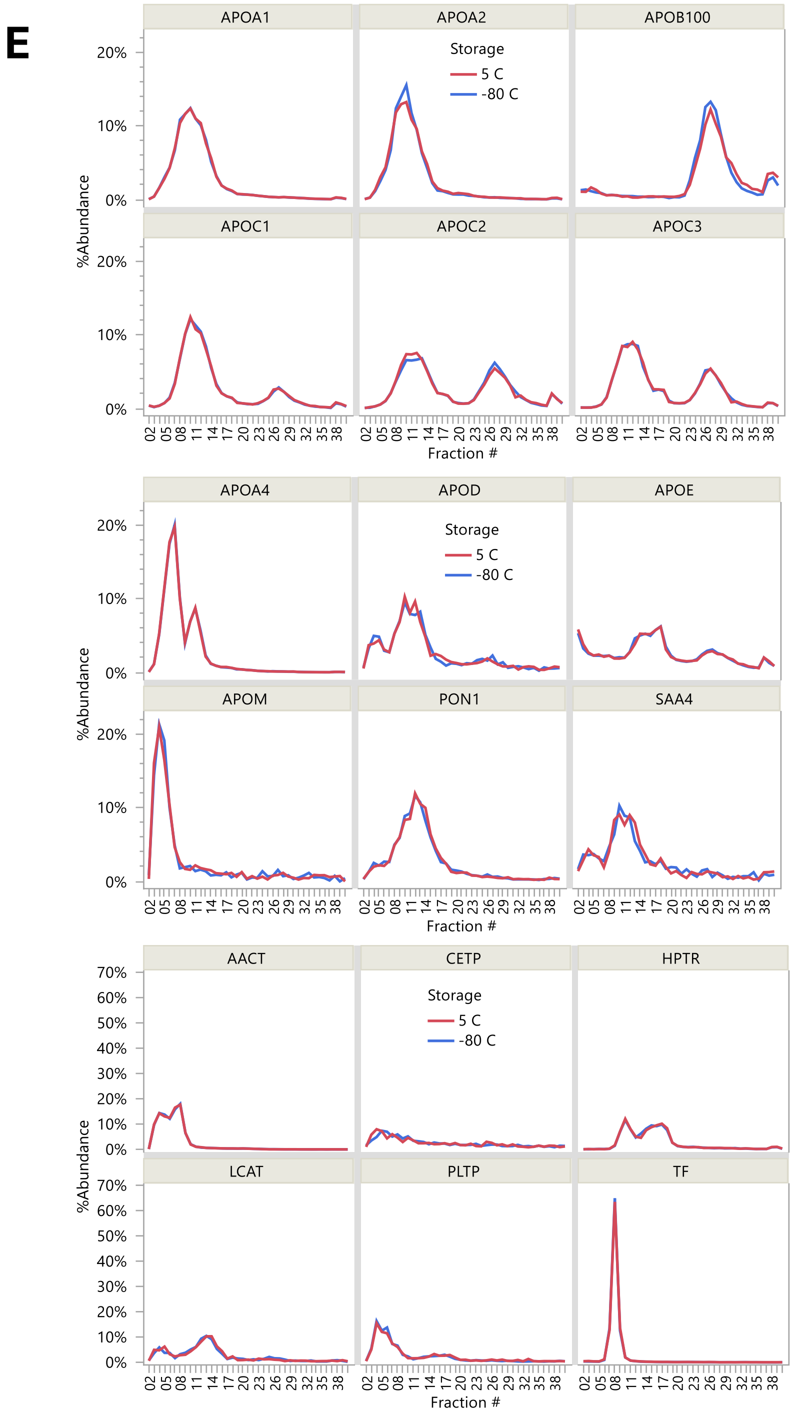

Figure S1 cont. Sample pool separated after storage at 5 °C and -80 °C for 1 day, analyzed on the same day in the same batch by AF4-LC-MS/MS workflow (E next page). One freeze-thaw cycle was a minor effect relative to other inter-day method variations (C), and differences between individual unknown samples (D).

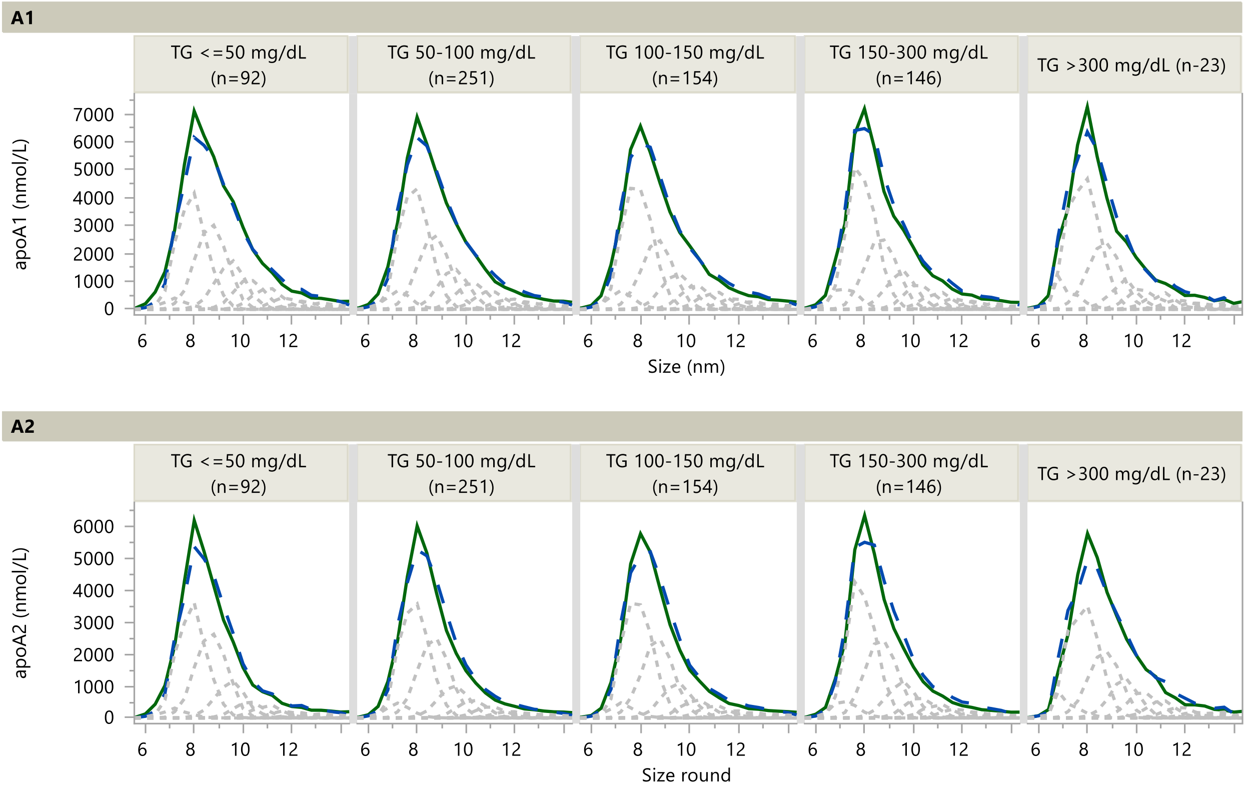

Figure S2. Deconvoluted apoA1 and apoA2 profiles stratified by total TG concentrations in the HDL size range. The green solid lines indicate non-deconvoluted data, and the blue dashed lines indicate the sum of deconvoluted Gaussians peaks and grey dashed lines indicate individual Gaussian peaks. Only the Gaussian peak that were in the 6-14 nm range are shown.

Note: To evaluate average concentration profiles, the size scale of was binned into 0.75, 1.5, and 3 nm increments in the HDL, LDL, and VLDL regions, respectively (1-2 fractions per bin). and the fraction concentrations were summed within each size bin. Similarly in Figures S3-S7.

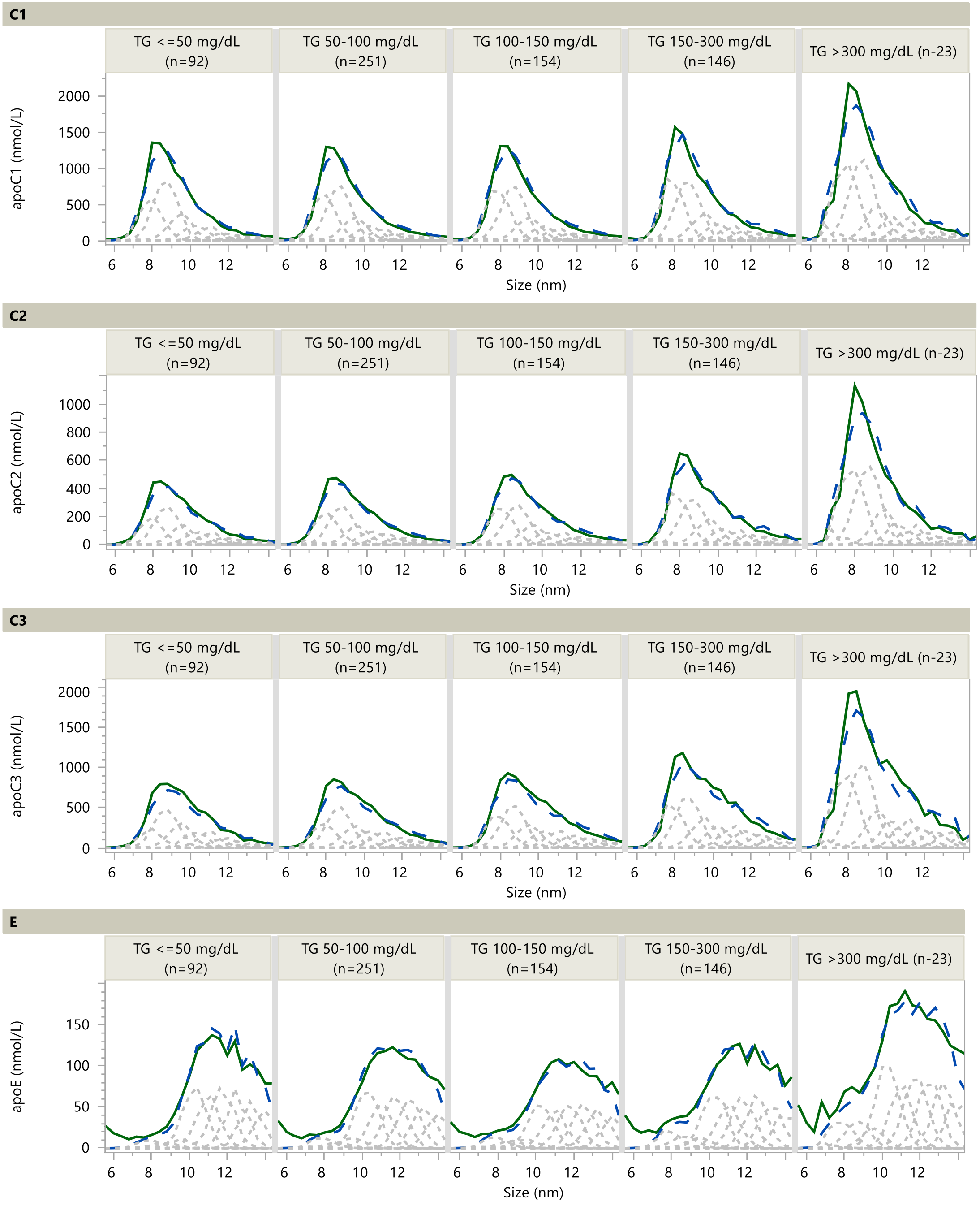

Figure S3. Deconvoluted exchangeable protein profiles stratified by total TG concentrations in the HDL size range. The green solid lines indicate non-deconvoluted data, and the blue dashed lines indicate the sum of deconvoluted Gaussians peaks, and grey dashed lines indicate individual Gaussian peaks. Only the Gaussian peak that were in the 6-14 nm range are shown. There is the greatest amount of noise at the TG<=50 mg/dL (n=92) and TG > 300 mg/dL (n=23) stratifications.

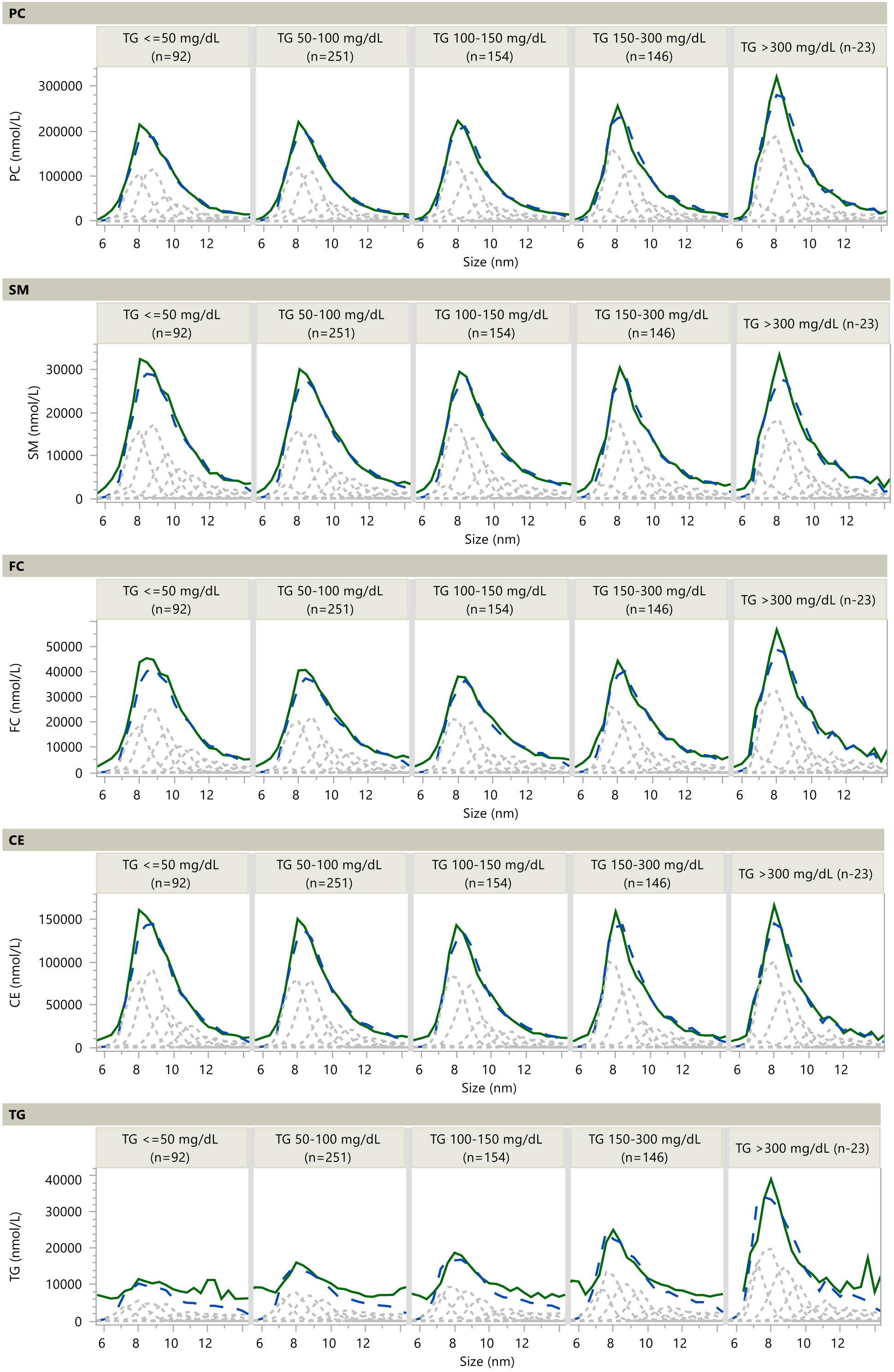

Figure S4. Deconvoluted lipid class profiles stratified by total TG concentrations in the HDL size range. The green solid lines indicate non-deconvoluted data, and the blue dashed lines indicate the sum of deconvoluted Gaussians peaks and grey dashed lines indicate individual Gaussian peaks. Only the Gaussian peak that were in the 6-14 nm range are shown. The most noise is depicted for the TG > 300 mg/dL (n=23) stratification.

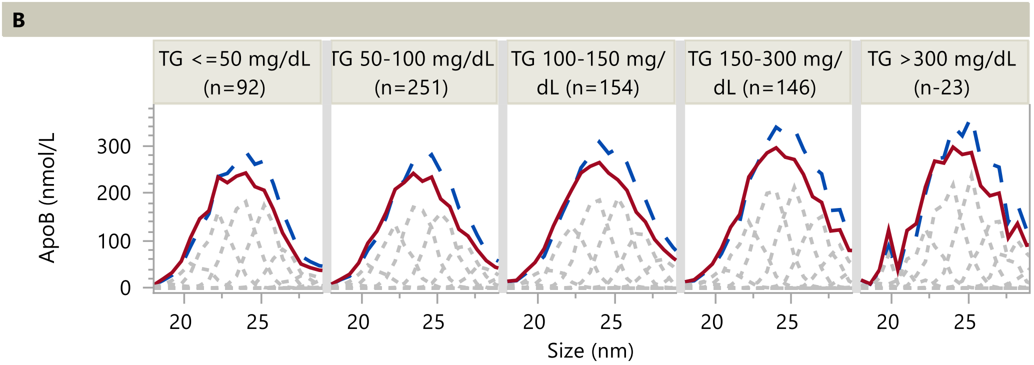

Figure S5. Deconvoluted apoB profiles are stratified by total TG concentrations in the LDL size range. The red solid lines indicate non-deconvoluted data, blue dashed lines indicate the sum of deconvoluted Gaussians peaks, and grey dashed lines indicate individual Gaussian peaks. Only the Gaussian peak that were in the.17-28 nm range are shown.

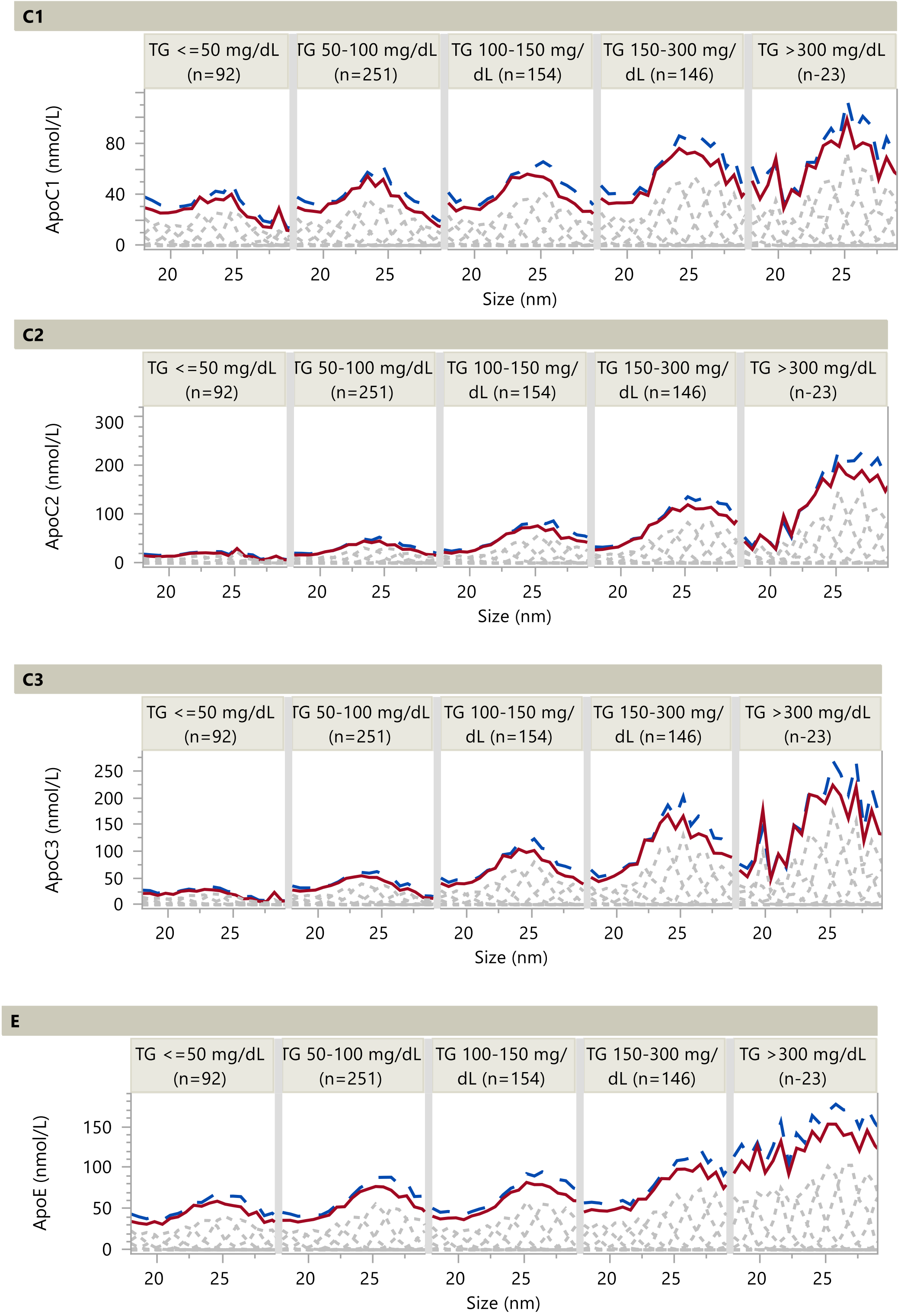

Figure S6. Deconvoluted lipid class profiles stratified by total TG concentrations in the LDL size range. The red solid lines indicate non-deconvoluted data, blue dashed lines indicate the sum of deconvoluted Gaussians peaks, and grey dashed lines indicate individual Gaussian peaks. Only the Gaussian peak that were in the.17-28 nm range are shown.

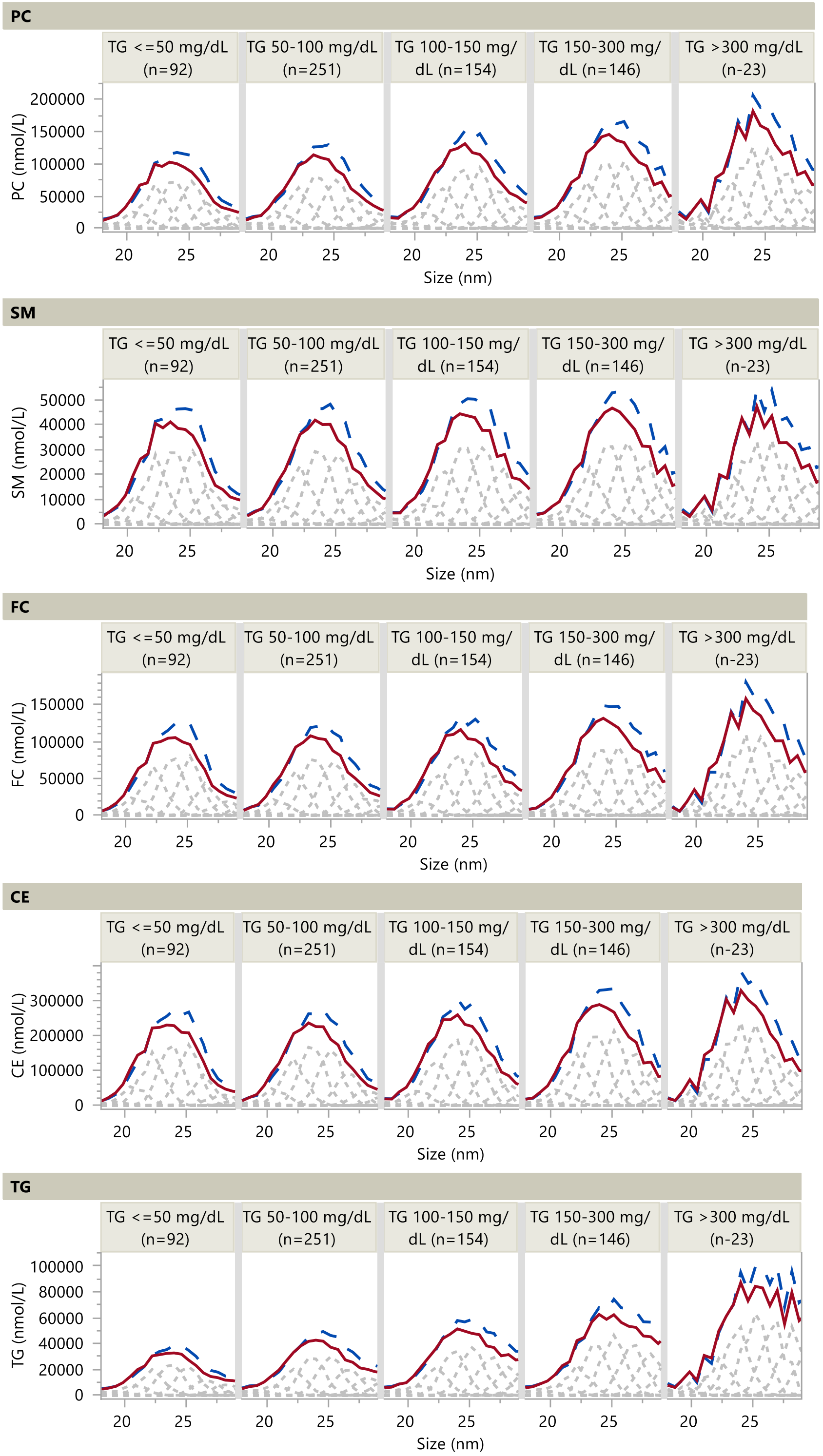

Figure S7. Deconvoluted exchangeable protein profiles stratified by total TG concentrations in the LDL size range. The red solid lines indicate non-deconvoluted data, blue dashed lines indicate the sum of deconvoluted Gaussians peaks, and grey dashed lines indicate individual Gaussian peaks. Only the Gaussian peak that were in the.17-28 nm range are shown.

A

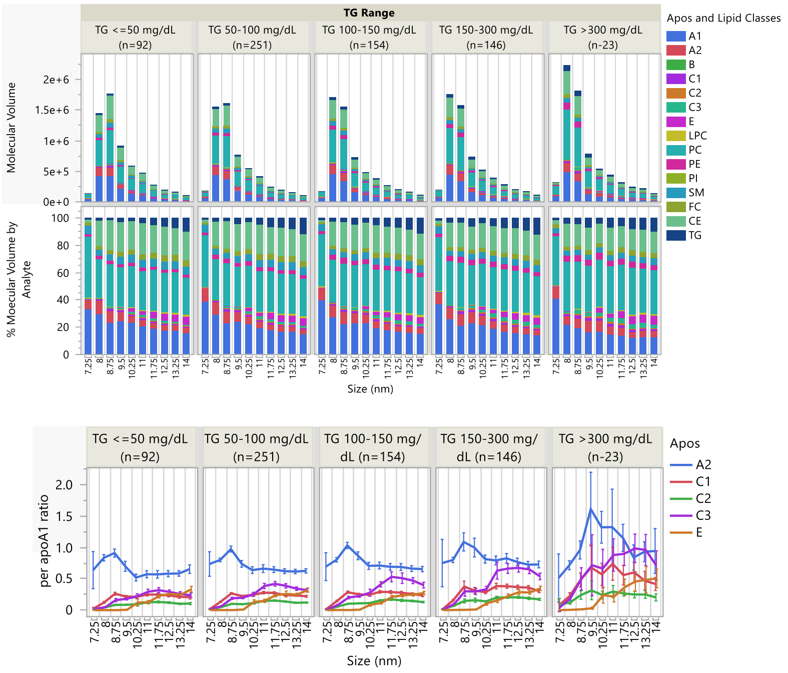

B

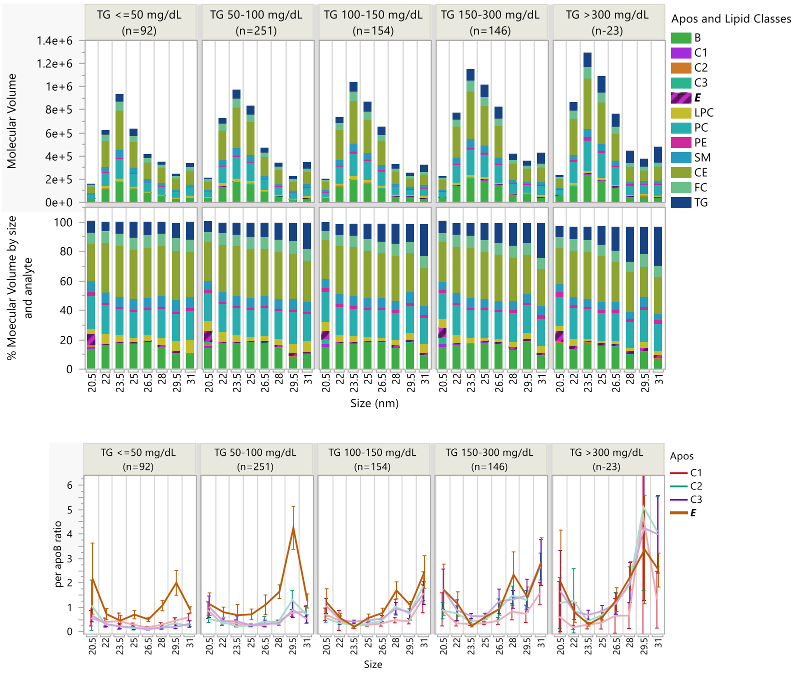

Figure S8. Absolute and relative molecular volume contributions to particle volume for HDL (A) and per apoB for LDL (B), after deconvolution of individual analyte profiles. Corresponding average number of apoA2, apoC1, apoC2, apoC3, and apoE per apoA1 (A, bottom). Corresponding average number of apoA2, apoC1, apoC2, apoC3, and apoE per apoB (B, bottom).

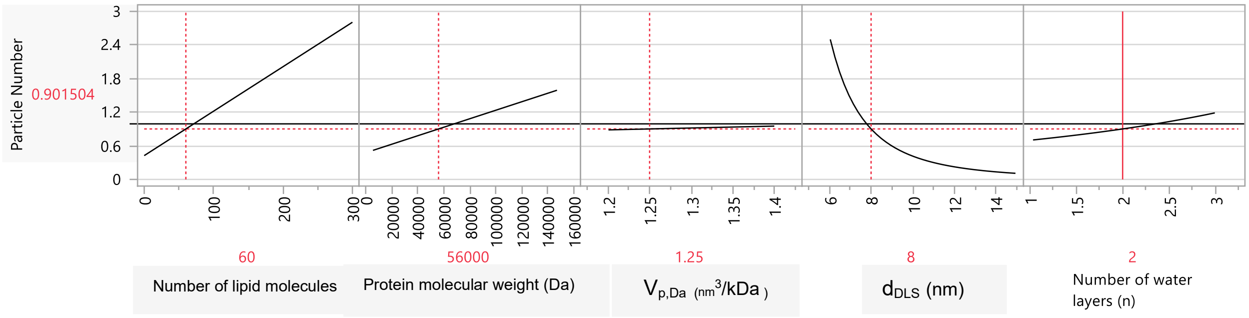

Figure S9. Simulation of the effect on particle number accuracy depending on number of lipid molecules (V_{lipid}=1.307 nm^3), variation in protein molecular weight, protein partial specific volume ($V_{p,Da}$), particle size (d_{DLS}), and number of water layers (w_h=0.3 nm). Note: Particle size impacts the most the particle number calculation. The partial specific volume has the least impact.

$$Particle\ Number\ (Lp\_P_m) = \frac{\left(N_{Lipid} * V_{Lipid} + + MW_{protein} * \ V_{p,Da}\right)}{d_{DLS} - \ 2n * w_h}$$

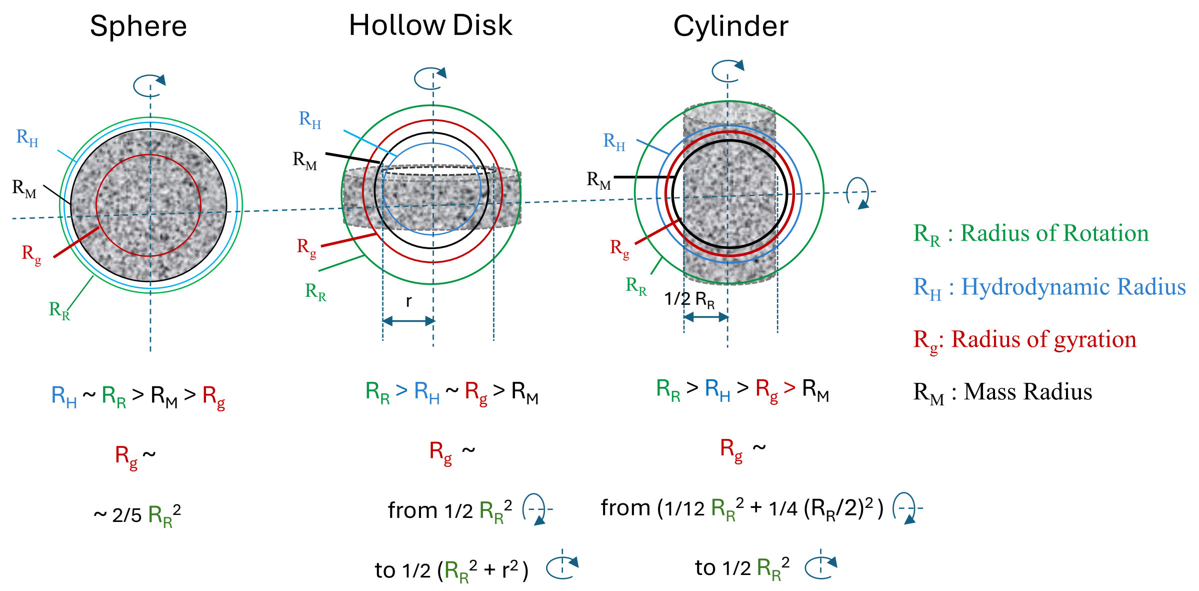

Figure 10. Characteristics of geometric particle shapes used in basic material sciences.

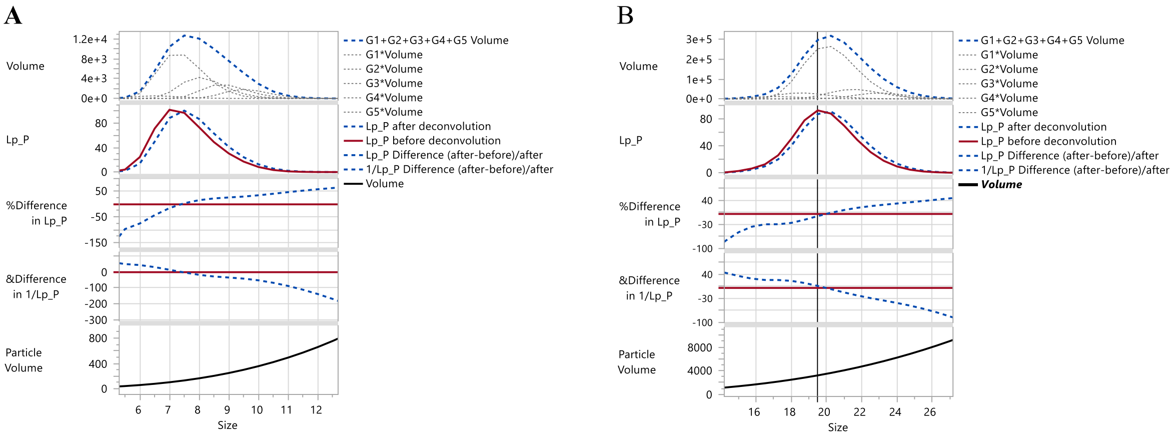

Figure S11. Simulation of particle volume and particle number profiles in the HDL size range **(A)** and the LDL size range **(B)**. Molecular volume (Volume) of five Gaussian subspecies, G1:G2:G3:G4:G5 with 5:60:20:10:5 relative intensities in the HDL size range **(A)** and with 5:10:70:10:5 relative intensities in the LDL size range **(B)**. Different particle number (Lp-P) were obtained by using the size measured in the fractions (continuous red line) or the size at the size maxima of each Gaussian subspecies (dashed blue line).

[Lp_P profile before deconvolution]: sum of Volumes at each size point on the y-axis then divided by the Size at the corresponding time point on the x-axis:

$$[Lp_P profile\ before\ deconvolution\ ] = \\ = \frac{[\,G_1 +\ G_2 +\ G_3 + G_4 +\ G_5]_{at\ size\ point}}{\frac{4}{3} * \pi * [\text{Size point}]^3}$$

[Lp_P profile after deconvolution]: sum of individual Gaussian components where the G_i-Size is the size at the center of the Gaussian component:

$$[Lp_P\ profile\ after\ deconvolution] = \\ = \frac{G_{1\ at\ size\ point}}{\frac{4}{3} * \pi *\ [\text{Size of } G_1]^3} + \frac{G_{2\ at\ size\ point}}{\frac{4}{3} * \pi *\ [\text{Size of } G_2]^3} + \frac{G_{3\ at\ size\ point}}{\frac{4}{3} * \pi *\ [\text{Size of } G_3]^3} + \frac{G_{4\ at\ size\ point}}{\frac{4}{3} * \pi * [\text{Size of } G_1]^3} + \frac{G_{5\ at\ size\ p[int}}{\frac{4}{3} * \pi *\ [\text{Size of } G_5]^3}$$

$$\%\text{Difference in Lp\_P} = \frac{[Lp{-}P\ profile\ after\ deconvolution] - [Lp\_P\ before\ deconvolution]}{Lp{-}P\ profile\ after\ deconvolution]} * 100$$

$$\%\text{Difference in 1/Lp\_P} = \frac{\frac{1}{[Lp{-}P\ profile\ after\ deconvolution]} - \frac{1}{[Lp\_P\ before\ deconvolution]}}{\frac{1}{Lp{-}P\ profile\ after\ deconvolution]}} * 100$$

Note, the %Difference varies depending on the relative abundance of the Gaussian subspecies, the size difference between subspecies, and the distance of the size point from the total size profile maxima.

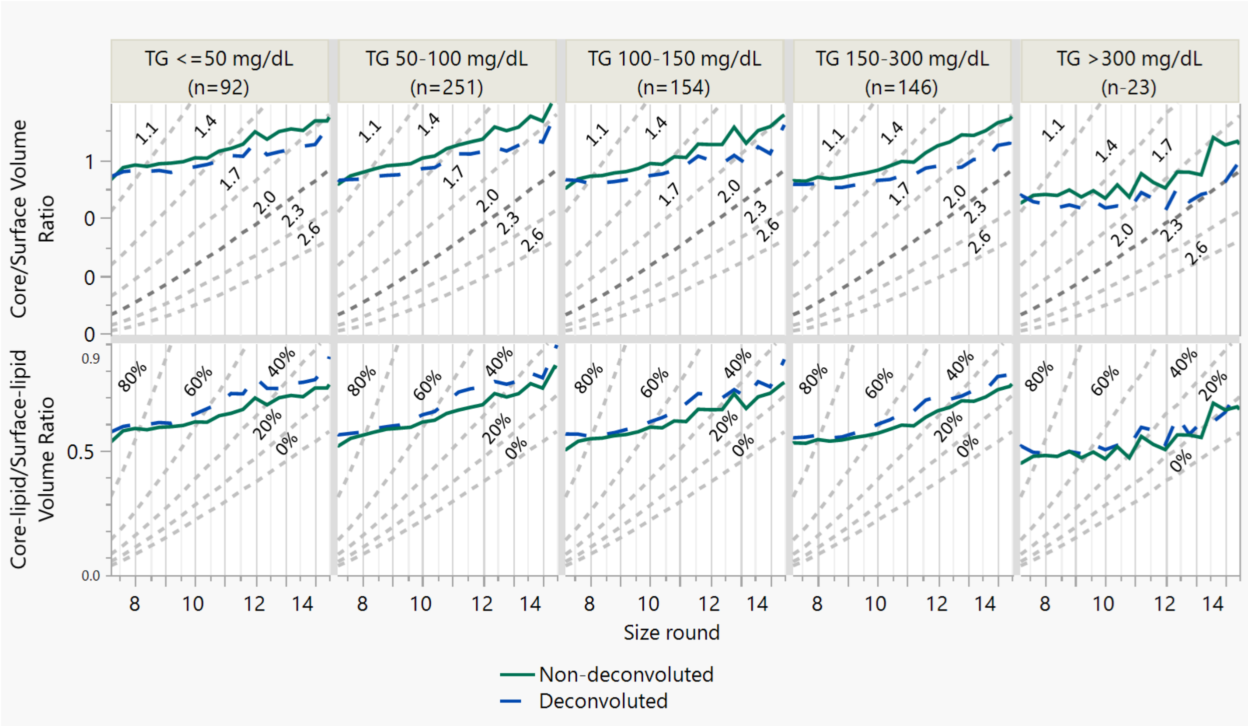

Figure S12. Core/surface and core-lipid/surface lipid volume ratios for HDL allowing estimation of the surface layer thickness (w_{SL}) and %protein volume in the 2 nm monolayer based on intercept with geometrically constrained expectations based on size of spherical particles. Green solid lines indicate raw experimental data, and blue dashed line indicate average of deconvoluted Gaussian peaks. Dashed lines indicate ratios using w_{SL} %protein in the monolayer as indicated by numbers. The graph is stratified by total TG concentrations in the HDL size range.

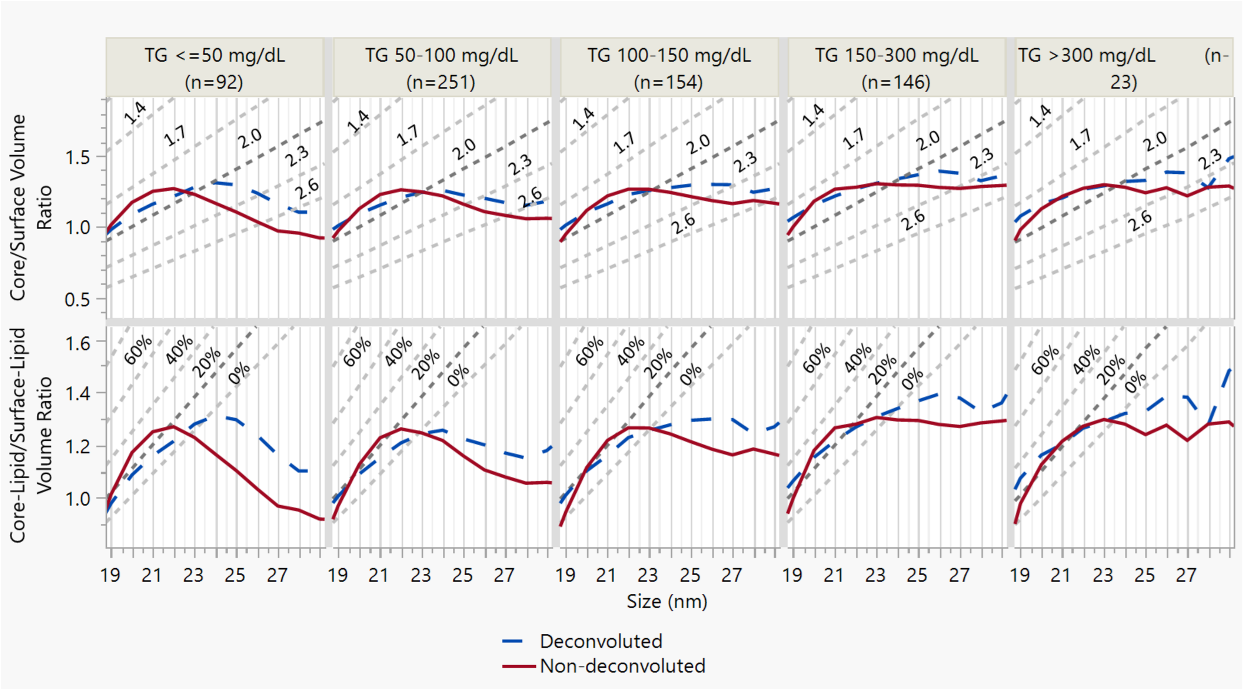

Figure S13. Core/surface and core-lipid/surface lipid volume ratios for LDL, allowing estimation of the surface layer thickness (w_{SL}) and %protein volume in the 2 nm monolayer based on intercept with geometrically constrained expectations based on size of spherical particles. Red solid lines indicate raw experimental data, and blue dashed line indicate average of the deconvoluted Gaussian peaks. Dashed lines indicate ratios using w_{SL} %protein in the monolayer as indicated by numbers. The graph is stratified by total TG concentrations in the LDL size range.

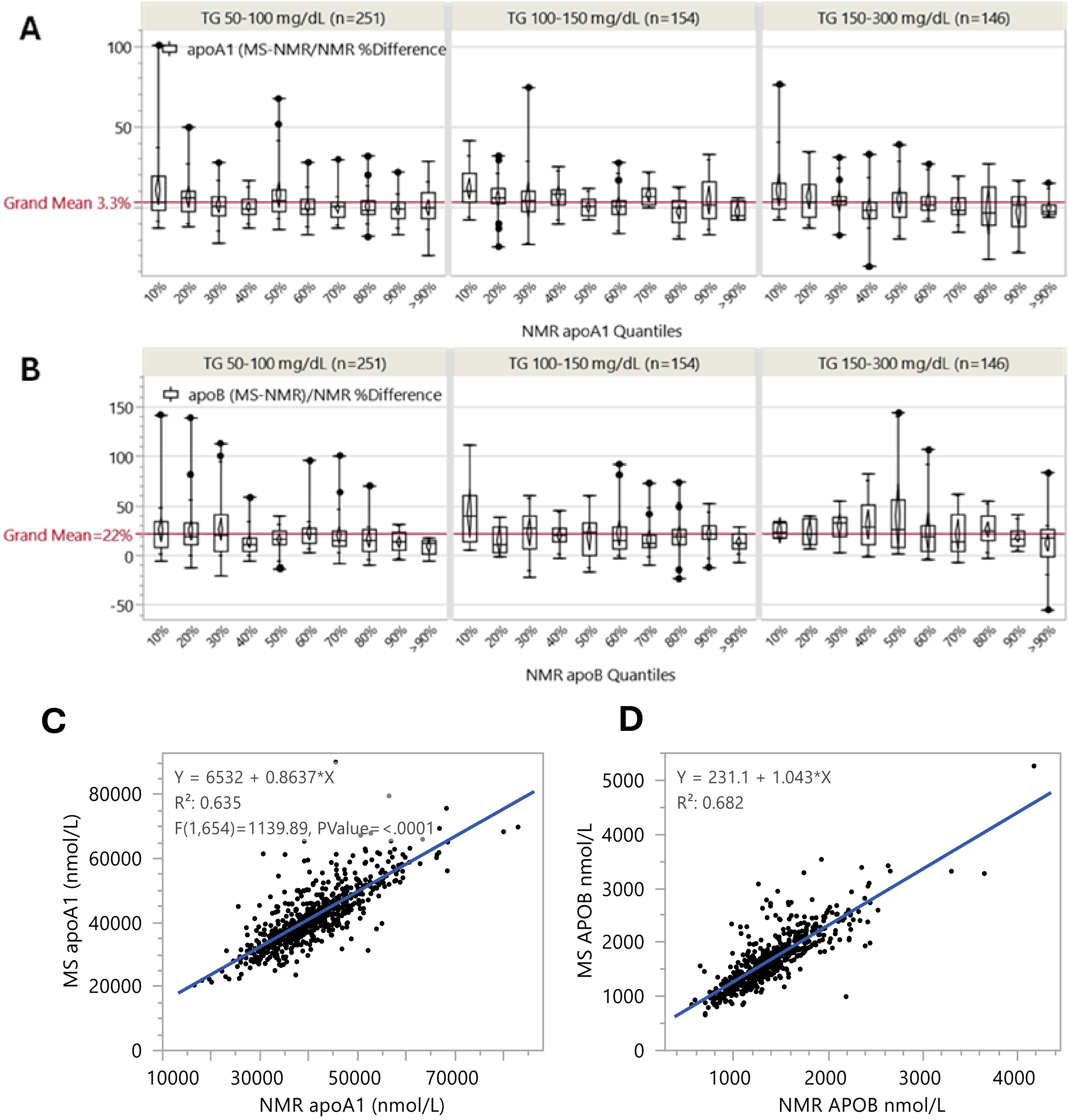

Figure S14. Comparison of total apoA1and apoB concentrations measured by LC-MS/MS and NMR. (A) Differences in total apoA1 (nmol/L) between LC-MS/MS and NMR by NMR quantiles. (B) Differences in apoB (nmol/L) between LC-MS/MS and NMR by NMR quantiles. (C) Correlation of total apoA1 by LC-MS/MS vs. NMR. (D) Correlation of total apoB by LC-MS/MS vs. NMR.

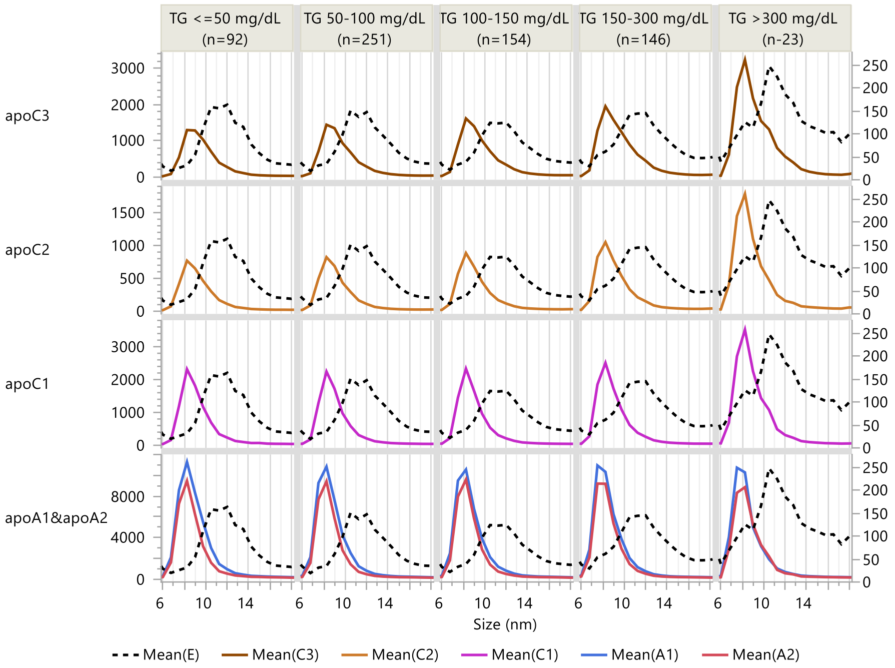

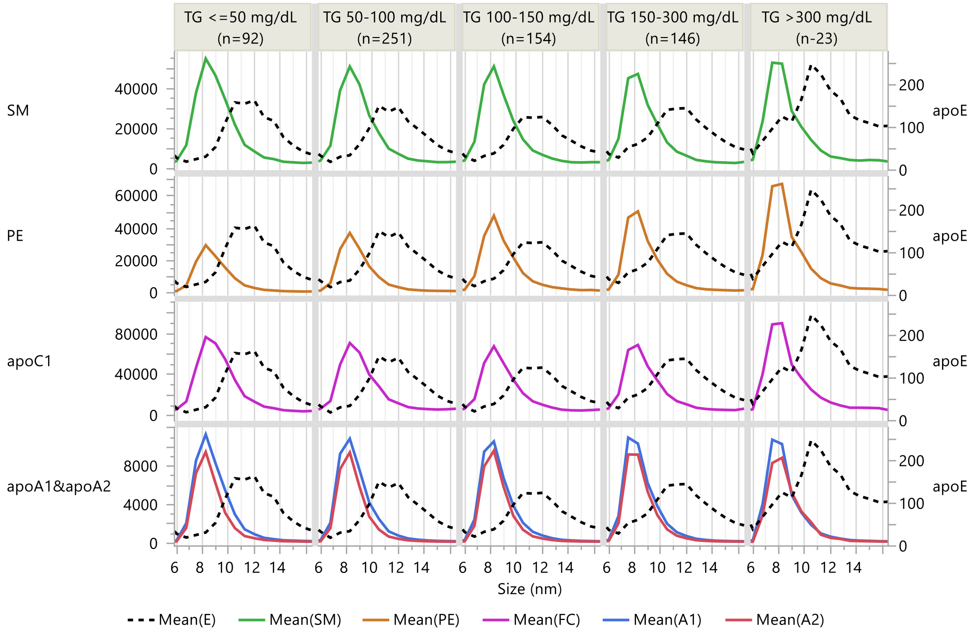

Figure S15. Evidence for the presence of distinct subpopulation of apoE-containing HDL particles. Overlay of average profiles of apoE (dotted line corresponding to right scales) with other HDL proteins and lipids (solid lines corresponding to left scales), stratified by total TG levels. Note: Profiles shown prior to deconvolution. ApoE profile maxima is at larger size by ~3-4 nm compared to apoCs and apoAs. At 7-8 nm (small HDL), there was an increase in fraction concentration of apoE across increasing total TG ranges. At 14-16 nm (large HDL), apoE presence increased while the apoC, apoA and lipid profiles decreased.

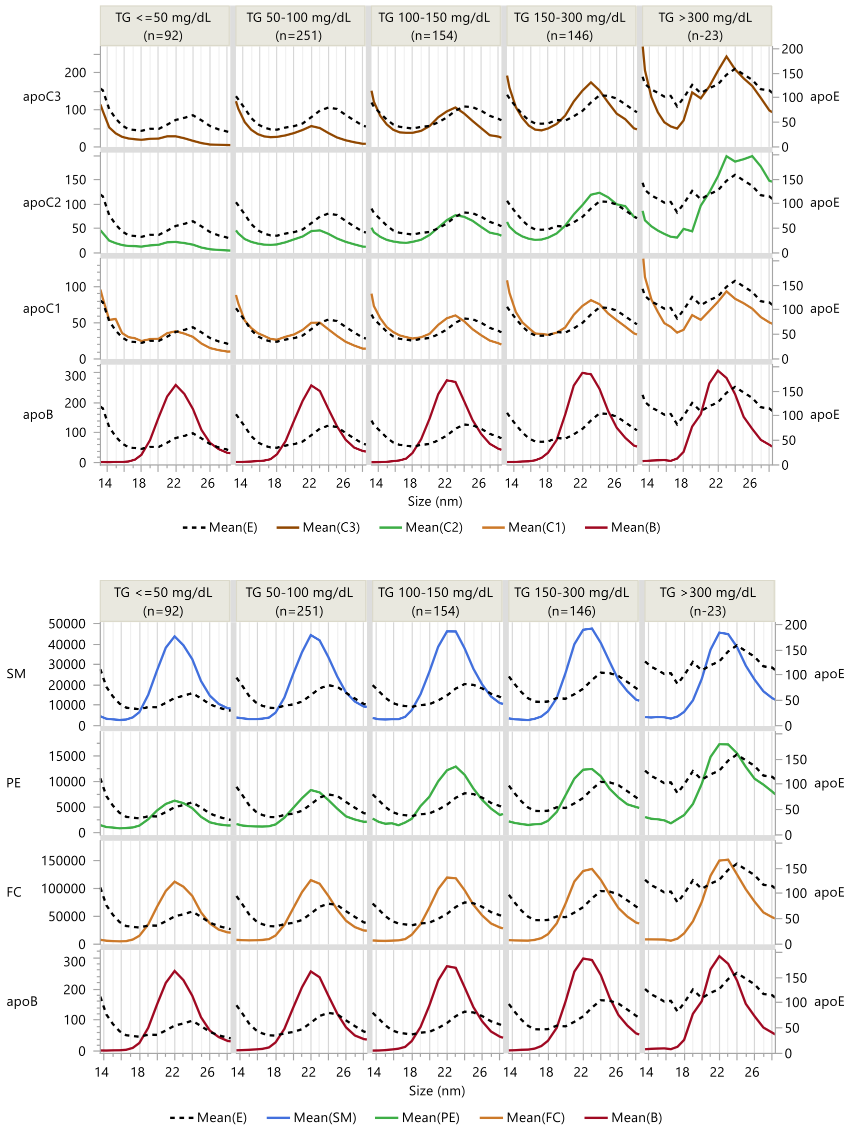

Figure S16. Evidence for the presence of distinct subpopulation of apoE-containing LDL particles. Overlay of non-deconvoluted, average profiles of apoE (dotted line corresponding to right scales) with other LDL proteins and lipids (solid lines corresponding to left scales), stratified by total TG levels. Note: ApoE profile maxima is larger in size compared to apoCs and apoB by ~2 nm. At 18-20 nm (small LDL), there was increase in fraction concentrations of apoE across increasing total TG ranges. At 22-28 nm (large LDL), apoE was present apoE while apoCs, apoB and lipids decreased.

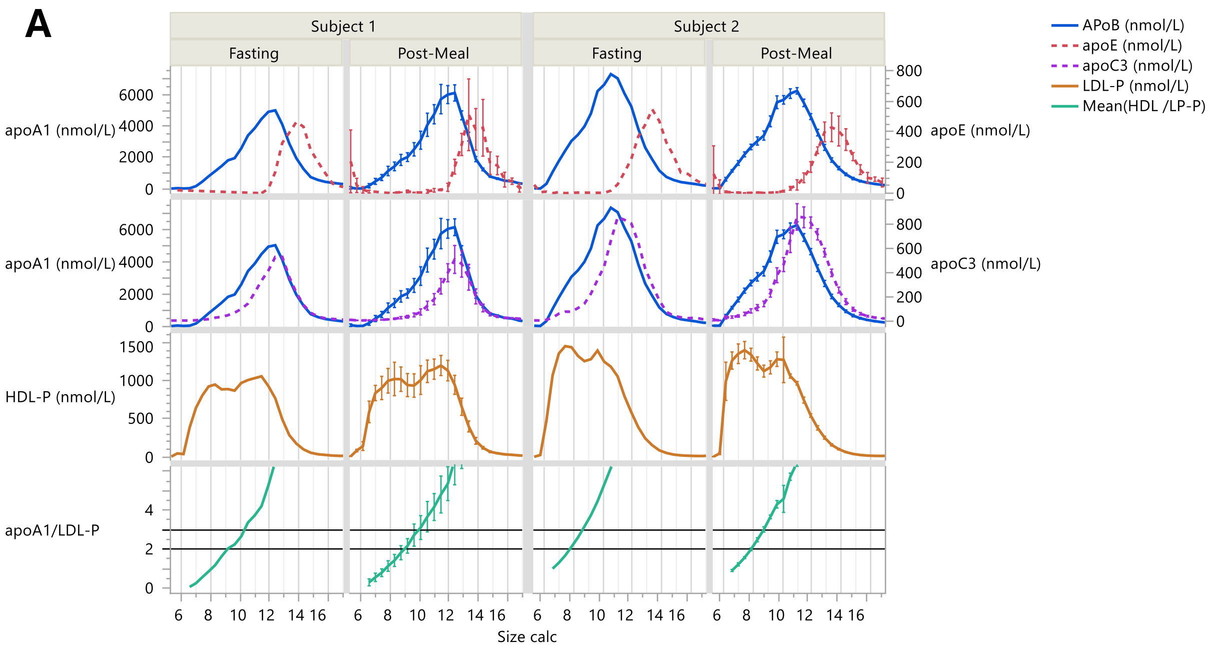

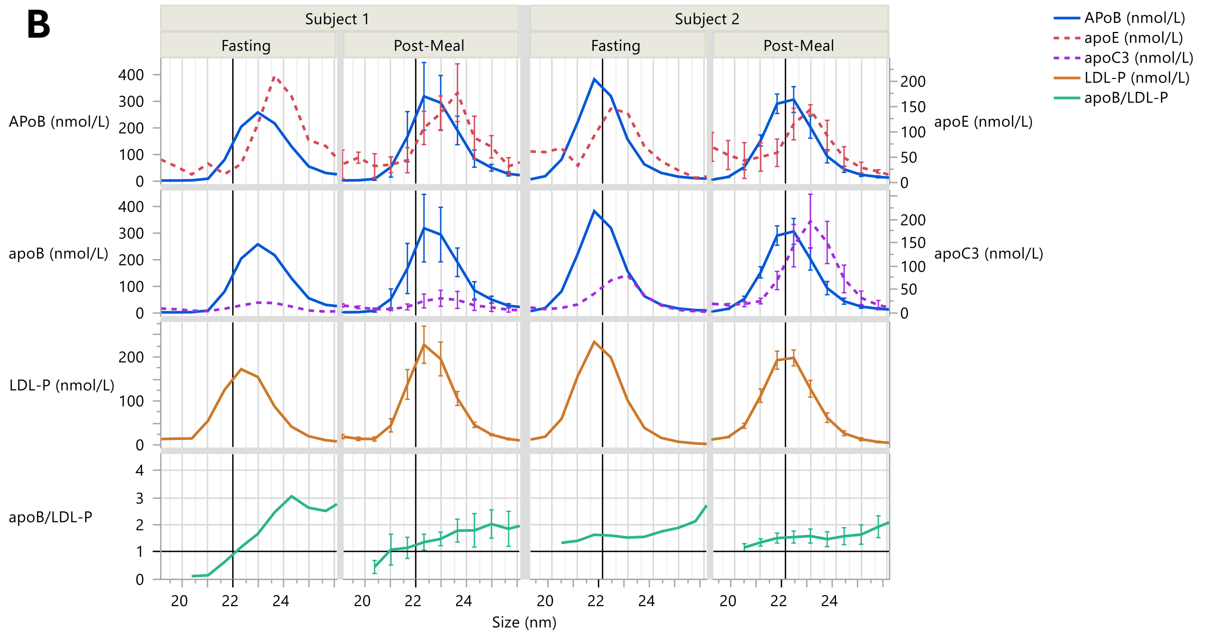

Figure S17. Observation of apoA1/HDL-P < 2 for S-HDL and apoB/LDL-P > 1 for L-LDL in freshly collected samples. Samples were drawn from two subjects after 10-hour fasting and multiple samples within 6 hours of a standardized fatty drink. (A) Overlay of apoA1 profiles with apoE and apoC3 (top) and estimated HDL-P and apoA1/HDL-P (bottom). (B) Overlay of apoB profiles with apoE and apoC3 (top) and estimated LDL-P and apoB/LDL-P (bottom).
